# A randomised trial of social support group intervention for people with aphasia: A Novel application of virtual reality

**DOI:** 10.1371/journal.pone.0239715

**Published:** 2020-09-24

**Authors:** Jane Marshall, Niamh Devane, Richard Talbot, Anna Caute, Madeline Cruice, Katerina Hilari, Gillian MacKenzie, Kimberley Maguire, Anita Patel, Abi Roper, Stephanie Wilson

**Affiliations:** 1 Centre for Language and Communication Science Research, City, University of London, London, United Kingdom; 2 School of Health and Social Care, University of Essex, Colchester, United Kingdom; 3 Anita Patel Health Economics Consulting Ltd, Queen Mary University of London, London, United Kingdom; 4 Centre for Human Computer Interaction Design, City, University of London, London, United Kingdom; IRCCS E. Medea, ITALY

## Abstract

About a third of strokes cause aphasia, or language loss, with profound consequences for the person’s social participation and quality of life. These problems may be mitigated by group social support. But this intervention is not available to all individuals. This study investigated whether it is feasible to deliver group social support to people with aphasia via a multi-user, virtual reality platform. It also explored the indicative effects of intervention and the costs. Intervention aimed to promote wellbeing and communicative success. It enabled participants to form new social connections and share experiences of living with aphasia. It comprised 14 sessions delivered over 6 months and was led by community based co-ordinators and volunteers. Feasibility measures comprised: recruitment and retention rates, compliance with intervention and assessment of treatment fidelity. Effects of intervention were explored using a waitlist randomised controlled design, with outcome measures of wellbeing, communication, social connectedness and quality of life. Two intervention groups were randomised to an immediate condition and two were randomised to a delayed condition. The main analysis explored scores on the measures between two time points, between which those in the immediate condition had received intervention, but those in the delayed group had not (yet). A comprehensive approach to economic data collection ensured that all costs of treatment delivery were recorded. Feasibility findings showed that the recruitment target was met (N = 34) and 85.3% (29/34) of participants completed intervention. All groups ran the 14 sessions as planned, and participants attended a mean of 11.4 sessions (s.d. 2.8), which was 81.6% of the intended dose. Fidelity checking showed minimal drift from the manualised intervention. No significant change was observed on any of the outcome measures, although the study was not powered to detect these. Costs varied across the four groups, from £7,483 - £12,562 British Pounds Sterling ($10,972 - $18,419 US dollars), depending on travel costs, the relative contributions of volunteers and the number of hardware loans that were needed. The results suggest that a larger trial of remote group support, using virtual reality, would be merited. However the treatment content and regime, and the selection of outcome measures should be reviewed before conducting the trial.

**Trail registration:** Study registered with ClinicalTrials.gov; Identifier: https://www.ncbi.nlm.nih.gov/NCT03115268.

## Introduction

Aphasia is an impairment of language caused by stroke or other neurological events, with profound consequences for a person’s well-being and quality of life. People with aphasia report reduced social networks [1], lower levels of social activity than their age matched peers [2] and lower quality of life than stroke survivors without aphasia [3]. Depression is a further risk, with rates in aphasia again exceeding the general stroke population [4]. Losses in social participation, coupled with the communication constraints imposed by aphasia, may threaten a person’s sense of self, leading some to describe aphasia as identity theft [5].

Despite the poor prognostic findings there are reports of many individuals living successfully with aphasia [6], suggesting that negative impacts are not inevitable or can be mitigated. It has been argued that group therapy may particularly contribute to this outcome, for example because group members can support one another and share ways of adjusting to aphasia [7]. Groups are a platform for natural communication and humour [8] and a potential forum for increasing social connectedness [9] and rebuilding a sense of self [7, 10].

Findings from a systematic review highlight the value of group therapy for people with aphasia [11]. The strongest evidence relates to benefits for language and communication, e.g. [12, 13]. However, there are also reports of enhanced social participation as a result of group intervention [9] and positive changes in conversational experiences [14]. Three studies, two of which were published after the review, report gains on measures of quality of life [15–17]. Despite these findings, the evidence that group therapy affects well-being in aphasia is not strong. Many studies did not include a control group [e.g.: 14–17] and two had fewer than ten participants [14, 15]. There are also studies that failed to detect significant change on a range of mood, well-being and identity measures following group intervention [18]. More positively, personal accounts from those who received group therapy suggest that groups can promote feelings of well-being [19] and lead to positive changes in self-identity [20].

When clinicians, policy makers and people with aphasia were consulted about best aphasia practice, they agreed that group intervention should be included in rehabilitation, particularly for those with persistent and long term aphasia [21]. However, this recommendation may not be realised in practice. For example, a survey of speech and language therapy provision in Australia found that 86% of community services were offering individual therapy, compared to just 36% who were offering group therapy [22]. A further study explored barriers to group provision [23]. These included resourcing and logistical barriers, such as a lack of funding, staffing constraints, and problems with transport. A commonly cited difficulty concerned the composition of groups, with respondents reporting that they had too few clients or clients with too disparate needs to form successful groups.

Using different models for delivering group provision might help to overcome some of these barriers. In the UK, many social support groups for people with aphasia are run by charitably funded organisations, often with the help of volunteers [24]. These provide an addition or alternative to National Health Service (NHS) input, particularly for those with chronic aphasia who have been discharged from speech and language therapy. As argued in a Kings Fund Report, there are many rationales for enlisting the support of volunteers in health care [25]. In addition to financial benefits the report cites evidence that volunteer services can improve wellbeing and reduce social exclusion amongst service users, such as people with mental health difficulties [26]. A study of volunteer run social groups for people with aphasia suggested that they helped patients to regain social interests and activities [27], although these conclusions were based on comparisons with a small and poorly matched control group [28].

While voluntary aphasia support groups are available in many UK settings, they may not be accessible to all. For example, Lanyon and colleagues [29] identified several barriers to group attendance that related to physical distance and the need to travel. These obstacles may be overcome by the use of telerehabilitation, which involves the remote delivery of therapy via digital technology. Thus the patient remains in their own home while the person delivering the intervention works from their clinical base or any setting with an internet connection. Telerehabilitation can reach individuals who live in remote areas or who are unable to travel. It also allows for recruitment over a wide geographical area, which may help with the problem of assembling viable groups.

There is growing evidence that telerehabilitation can be used to both assess [30, 31] and treat [32–34] people with aphasia. While most applications of telerehabilitation have been delivered one to one, researchers in Australia have additionally explored the feasibility of delivering remote group therapy [35–37]. This team developed a 12 week (18 hour) programme of therapy called TeleGAIN, which aimed to improve communication related quality of life. Topic based sessions, for example focussing on travel and hobbies, provided opportunities for communication exchange, for sharing personal life histories and for mutual support in living with aphasia. The therapy was delivered to groups of four participants using an Adobe Connect video conferencing platform. A pilot study and mixed methods phase 2 trial showed that TeleGAIN was feasible [35] and positively perceived by those who delivered the intervention [37]. Those who received TeleGAIN made significant improvements on a range of measures, including the Assessment of Living with Aphasia and the Comprehensive Aphasia Test [36]. However, the lack of a control group means that these gains cannot be attributed unambiguously to the therapy.

The study reported in this paper further explores the feasibility of remote provision of group social support for people with aphasia. The delivery platform was EVA Park, a multi-user virtual world designed with and for people with aphasia [38]. EVA Park contains a number of functional locations, such as a café and hair salon, where users can practise everyday language. There are also fantasy settings, such as a tardis (based on the UK television series ‘Dr Who’) which transforms into a gaming hall when entered. These were designed to stimulate amusement and conversation. Users are represented by personalised avatars and communicate in real time mainly via speech (there is also a message typing facility). EVA Park can be accessed from a user’s home, on a medium specification computer with a reliable internet connection.

Previous research has shown that EVA Park can be used to deliver language stimulation to people with aphasia, with significant benefits for functional communication [39]. Participants were also very positive about the experience of using EVA Park [40] and the platform supported a range of communicative interactions [41]. Subsequent studies have documented successful use of the platform for targeted language therapies [42, 43].

Applications of EVA Park to date have mainly involved one-to-one therapy and support. However, a number of factors highlight its potential to host group interventions. It is a multi-user platform and contains attractive places where groups can meet. For example, there is a tree house with a ring of cushion seats, a café, an outdoor tropical bar, and a lakeside setting with multiple recliners. The first EVA Park study employed mainly individual therapy, but included one group session per week [39]. A linked Human Computer Interaction study showed that these group sessions were increasingly valued as therapy progressed. For example, in the final week participants gave a mean satisfaction rating of 4.2, out of a maximum score of 5, for the group sessions [41]. Interviews with users indicated that some found the groups challenging, but many also commented on the positive support and humour that derived from meeting others with aphasia in EVA Park [40].

This study aimed to explore whether EVA Park could successfully host a programme of remote support group intervention. The study extends previous findings with EVA Park by exploring a different form of intervention that was entirely group based. The therapy also targeted well-being as a primary outcome, in contrast to previous research where the focus was on communication [39] and language [42]. The intervention in the current study was delivered across four groups and was designed to resemble a typical, UK model for group social support. It was non-intensive, delivered from a community context, with each group led by a local co-ordinator and supported by volunteers. The study aimed to inform a potential future trial of this intervention and provide data for service providers, who might adopt this model of group delivery. Data were also collected, therefore, on the cost of the intervention.

The study addressed the following questions:

RQ1 Is delivery of group social support to people with aphasia feasible via EVA Park, as indicated by the recruitment and retention of participants, compliance with intervention, and fidelity assessments of intervention?

RQ2 Does a programme of group social support delivered in EVA Park improve scores on measures of well-being, communication, social connectedness, language and quality of life?

RQ3 What is the cost of providing a non-intensive programme of group social support delivered in EVA Park?

This work formed part of a mixed methods feasibility trial. Additional interview, consensus discussion and observation data were collected to examine the acceptability of the intervention and participants’ experiences with EVA Park. These qualitative findings will be reported in subsequent papers.

## Method

This study was granted ethical approval by the Ethics Committee of the School of Health Sciences, City, University of London (Language and Communication Science Proportionate Review) LCS/PR/Staff/16-17/06. All participants (group co-ordinators, group volunteers, group participants with aphasia) gave informed written consent. Where relevant, information and consent materials were designed to be accessible to people with aphasia [44]. The study was registered with ClinicalTrials.gov; Identifier: NCT03115268.

### Participants

Six group co-ordinators were recruited (4 female, 2 male), each of whom led one of the 4 intervention groups (two groups had paired co-ordinators working in a job share). All had at least 3 years’ experience, (mean 4.7. Range 3–7) in leading community groups for people with aphasia. One was a qualified speech and language therapist (SLT). Co-ordinators, and their associated groups, were geographically dispersed. For example, one was in the North of England and one was in the South. Groups were designated ‘North’, ‘South’, ‘East’ and ‘West’ according to their location.

Eleven volunteers were recruited (6 females, 5 males), with at least two assigned to each intervention group. Note that this was below our protocol target of 4 per group. Some had long-standing links with the co-ordinator and their respective community setting. Others were newly recruited for this study. All had previous experience in working with people who have aphasia. Four were experienced group volunteers, three were student speech and language therapists (SLT) and one was a qualified SLT. Three were stroke survivors, two of whom had mild aphasia.

Thirty-four people with aphasia caused by stroke were recruited by the project managers (Authors ND, RT). The selection criteria were as follows: moderate or mild aphasia; no co-existing diagnosis affecting cognition; no severe hearing or visual impairments; fluent in English before their stroke. Eligibility was established by administering the Frenchay Aphasia Screening Test [45] and a brief screening questionnaire, e.g. asking about the history of the stroke, language background and any co-morbidities. The criteria aimed to ensure that individuals would be able to participate in the intervention. For example, communication in EVA Park is largely speech dependent making it difficult to access by people with severe impairments in the production or comprehension of speech. Severe cognitive and sensory impairments also impede use of this technology. Computer competence was assessed with a Dynamic Assessment of Computer Learning [46, 47]. Here the individual was required to carry out a simple instruction using a laptop or tablet computer, such as consulting an internet weather report. If unable, they were provided with a hierarchy of cues, including verbal instructions and modelling, and their ability to respond to these cues was assessed. This ensured that the participant either had basic computing skills or could develop these with support. Prior computer usage was not an exclusion criterion.

Participants were either existing members of the co-ordinators’ community groups or were assigned to groups on the basis of their geographical location. This aimed to ensure that relationships formed during the online intervention might be sustained in the real world after that intervention ceased.

The sample size was informed by recommendations for feasibility studies which call for between 24 and 50 participants [48, 49]. The size of each intervention group was also constrained by the number of people who could be effectively managed in EVA Park. As this was a feasibility study, the sample was not powered to provide definitive data about the benefits of the intervention.

Participant recruitment began on 16.5.2017 (date of first screening appointment). Data collection ended on 17.11.2018 (date of final follow up assessment).

### Intervention

There were four intervention groups, each led by at least one co-ordinator and 2 volunteers, and each involving between six and nine people with aphasia. Intervention comprised 14 group sessions (21 hours) delivered over 6 months, with sessions occurring once a fortnight (see S1 Supporting Information in S1 File for an outline of sessions and sample session plans). All sessions were run remotely, in EVA Park. Thus participants accessed the intervention on a computer in their own home. Co-ordinators and volunteers worked either from a home computer or from a computer in their community centre. All participants were represented by personalised avatars in EVA Park, which were set up before the start of intervention.

The intervention was defined in a manual that drew on published accounts of support interventions for people with aphasia [e.g.: 50, 51]. The aims and content of the intervention were also informed by discussions with our Advisory Group, comprising four people with aphasia, a family member and three voluntary sector staff members who were responsible for managing and delivering stroke services. Intervention aimed to counter the negative impacts of aphasia on quality of life, and to facilitate living well with aphasia [6]. Activities aimed to promote wellbeing, give participants experiences of communicative success and foster social connection between group members. Group members frequently reflected on personal strengths, and how these were applied to living with aphasia. Issues of personal identity were also focussed, given the impact of aphasia on a person’s sense of self [5, 51].

Each intervention session was based on a topic, which was chosen with the Advisory Group to address the intervention goals. Several topics enabled group members to share experiences of living and coping with aphasia (e.g. ‘You’; ‘Aphasia’; ‘Resilience’; ‘Personal Strengths’). These sessions drew on the exercises described by Holland [52] that foster adaption, growth and the development of positive attitudes concerning aphasia and its consequences. For example, in the session on ‘Aphasia’ participants discussed their residual communication strengths and the strategies that they employed to ease communication.

Other topics aimed to stimulate social connection and positive communication exchanges. They provided opportunities for group members to express opinions and convey aspects of their personality, thus addressing the theme of identity. These topics included ‘Comedy’, ‘Music’, ‘Art’, ‘Literature’ and ‘Eating Out’. To illustrate, the Comedy session involved a discussion about the benefits of comedy for wellbeing, sharing views about comedy, reacting to a comedy video clip (displayed on a media screen in EVA Park) and sharing personal stories about funny events.

All sessions aimed to give participants the experience of communication success. Formally correct language was not demanded and total communication devices were encouraged, e.g. employing alternatives to speech [53]. These devices included tone of voice, message writing and demonstration. The EVA Park avatars were able to execute a small number of pre-programmed gestures (such as waving or an exaggerated belly laugh), which were performed when a screen icon was clicked. These were used to supplement communication, for example to express appreciation over a joke.

Another thread that ran through the intervention was the identification of personal strengths, and reflection on how participants used these in their daily lives. This was in line with the principles of positive psychology and asset-based interventions [54–56]. For example, each session included a review activity at the end in which participants were asked to identify ‘three good things’ that had taken place. Applying personal strengths in the context of meaningful activities was particularly addressed in two ‘Project’ sessions. Here, group members chose to create something collectively (e.g. an aphasia awareness film, or an audio podcast about aphasia). This encouraged the group to work towards a shared goal, and to draw on their individual skills in realising that goal. For example, one former video editor used his skills in making a film about EVA Park.

Group leadership was provided by the co-ordinators. They introduced each topic and led the activities, for example by assigning roles and turns to group members. They ran group discussions, ensuring that each member had the opportunity to contribute. They managed transitions between activities. They closed each session with feedback to the group and by looking ahead to the following week. Volunteers contributed to group discussions and supported individuals’ communication attempts, for example by asking questions or providing cues. They supported small groups or pairs of participants in instances when the groups sub-divided.

Between session challenge tasks (e.g. to meet with another group member for exercises in the EVA Park Health Centre) aimed to provide further experiences of success and extend the social contacts made in the groups. These tasks required group members to access EVA Park independently, without the support of volunteers.

### Training of the intervention providers

Before the intervention began, the project managers (ND, RT) provided the group co-ordinators and volunteers with two, 4-hour face-to-face training sessions. The first session covered technical aspects of group provision in EVA Park including how to set-up participants in EVA Park, avatar creation and customisation, navigation in EVA Park, troubleshooting and providing remote technical support to people with aphasia. The second session provided training on delivering the intervention described above. This included practice in managing a group in a virtual environment. Specific advice was given on how to employ supportive communication techniques in EVA Park. Such techniques included pointing, giving time, reflective listening, using message writing to support understanding, and using visual cues. In addition to training, the project managers provided monthly supervision sessions to the co-ordinators and volunteers from each intervention group (6 sessions per group, each 40 minutes long). These took place in a private area of EVA Park that was not accessible to all users. Sessions covered general feedback, attendance, technical issues and solutions, use of interactive elements and immersion in EVA Park. They also covered research administration such as record keeping and economic data collection. Support was also provided to co-ordinators and volunteers throughout the 6-month intervention period, through responses to telephone and e-mail queries.

### Training and set up of intervention recipients

The co-ordinators set up and trained their group participants, through individual home visits. Set up involved downloading the browser that ran EVA Park (Firestorm) onto their computer, adding the EVA Park web address, and setting up the participant’s avatar. Training followed a 20 minute protocol and taught each participant how to log in; make their avatar walk, fly and sit; turn on their microphone; adjust the volume of other users and adjust their camera angle.

EVA Park required an internet connection of at least 2MB per second download and 500KB per second upload. Recommended ‘standard’ computer requirements were: 1GHz processor, 1GB memory and a dedicated graphics card (such as Nvidia graphics cards 6600 or better). Twenty participants had hardware that did not meet these requirements. They were, therefore, loaned a computer for the duration of their intervention.

### Design

The study employed a randomised, waitlist controlled design. Two intervention groups were randomised to run in months 6–11 of the study (immediate condition). The other two groups ran in months 13–18 (delayed condition). Randomisation took place before any participants with aphasia were recruited and was conducted using a computer randomisation tool (the list randomiser in www.random.org). Those who delivered the intervention (co-ordinators and volunteers) and those who received intervention (the participants with aphasia) were not blinded to condition. Those who administered and scored the outcome measures were blinded (see below). Participants only had access to EVA Park during their intervention period. Usual care continued for all participants with aphasia for the duration of the study.

### Feasibility measures

The number of participants screened, recruited and retained during the study was recorded, together with reasons for non-recruitment and attrition. Records of attendance at intervention were kept. Automatic computer logging recorded the number of times EVA Park was visited and the amount of time spent in EVA Park by each participant. The number of adverse events experienced by participants was recorded.

We aimed to record 30 intervention sessions using screen capture technology (3 recordings failed owing to technical difficulties). Sampling was spread across the groups and ensured that sessions were filmed from early and late weeks of the intervention. The sampling regime was drawn up blind to the content of intervention and before sessions were run. Eighteen of the recorded intervention sessions (just over 32% of all intervention sessions) were subject to fidelity checking. A fidelity checklist was developed with reference to the intervention manual and in discussion with the project managers (ND & RT). This reflected core features of the intervention, or elements that were essential to the underlying principles of the intervention (see S2 Supporting Information in S1 File). The recorded sessions were reviewed, and each feature on the checklist was scored as fully present (2), partially present (1), absent (0) or not applicable. The fourth category covered instances in which there was no opportunity for a feature to arise in a session. Fidelity scoring was conducted by two researchers who were not involved in delivering the intervention or in any other aspect of this study (Authors GM, KM). Both were qualified speech and language therapists. Five sessions were scored independently by both researchers to assess inter-rater reliability. As there were 24 items on the fidelity checklist this yielded 120 double coded treatment components.

### Outcome measures

Two primary outcome measures were identified:

Warwick-Edinburgh Mental Well-being Scale (WEMWBS) [57]. This scale was developed to monitor mental wellbeing in the general population and evaluate intervention effects on mental wellbeing. It comprises 14, positively worded statements, such as ‘I’ve been feeling good about myself’, that have to be rated on a 5 point scale. These are summed, yielding a single wellbeing score (/70).

Communication Activities of Daily Living-2 (CADL-2) [58]. This is a standardised assessment of everyday communication for people with aphasia, which is based on specific scenarios, such as going to the doctor. The CADL-2 contains 50 items scored 2, 1 or 0. Scoring credits communicative success rather than the use of formally correct language. The total score was analysed; i.e. the score across all 50 questions (/100).

Secondary measures were:

Social Connectedness Scale-Revised (SCS-R) [59]. This consists of 20 positively or negatively worded statements, such as ‘I feel close to people’ and ‘I see myself as a loner’, that have to be rated on a six point scale. All items are summed (with reverse scores for negative items) to produce a single score (/120).

Western Aphasia Battery-Revised (WAB-R) [60]. This is a standardised language assessment designed for people with aphasia. Only sections assessing speech production and comprehension were administered. These produce a single aphasia quotient score (/100) that was analysed here.

Stroke and Aphasia Quality of Life-39 (SAQOL-39g) generic version [61]. In this assessment 39 items, such as asking how much trouble someone had speaking in the last week, must be rated on a five- point scale. Three domains are covered: physical, psychosocial and communication, yielding mean domain and total scores. The mean total was analysed here (/5).

All measures have strong psychometric qualities and have shown sensitivity to therapy induced change. Three (CADL-2 [58], WAB-R [60] and SAQOL-39g [61]) have been widely used in previous trials of aphasia therapy [62], and two (SAQOL-39g [61] and WAB-R [60]) have since been advocated as core outcome measures, for use in all aphasia therapy trials [63]. Two measures (WEMWBS [57] and SCS-R [59]) were not designed and have not been validated for people with aphasia. However, they are brief and make limited language demands. The WEMWBS [57] has been used in a trial of peer support for people with aphasia [64]

The outcome measures were administered to participants with aphasia in months 5 (T1), 12 (T2) and 19 (T3). Thus, participants in the immediate condition were tested before intervention, after intervention and at 6 months follow up. Those in the delayed condition were tested twice before intervention began and once after intervention. Testing was conducted face to face, not in EVA Park. Participants were tested in their own homes, or in their local community group setting. Testers were not involved in any other aspect of the study, and were blinded to time point and condition. Testers were qualified or student speech and language therapists who were trained in test administration.

Mean scores, standard deviations (s.d.) were calculated at each time point. For skewed variables, medians and interquartile ranges (IQR) were also calculated. The primary analysis employed ANCOVA to compare assessment results at T2 across participants in the immediate and delayed conditions, with scores at T1 as the covariate. Here, indicative treatment effects would be demonstrated by a significant group effect favouring those in the immediate condition. This analysis was performed on both an intention to treat (ITT) and per protocol basis. The former used carried forward scores for any missing data. A secondary, per protocol, analysis examined pre to post intervention change across all participants who completed the intervention and for whom outcome measure data were available.

### Economic data collection

A comprehensive and detailed approach was taken to assess economic costs for each group from a provider perspective. This entailed (a) identifying all key inputs associated with the intervention, (b) devising approaches and instruments to measure such inputs separately for each group, (c) estimating the costs associated with all inputs separately for each group, (d) estimating total costs separately for each group and average total costs across the four groups and (e) examining variations in costs across the four groups.

The following key input components were identified and then measured either prospectively (by researchers) or retrospectively (by coordinators and volunteers) using a combination of specifically designed forms issued online and routine project records:

- resources and expenses associated with the project managers delivering training for coordinators and volunteers and supporting the running of the groups;
- resources and expenses associated with service coordinators attending training and contributing to the running of the groups;
- resources and expenses associated with volunteers attending training and contributing to the running of the groups;
- loaning of computer hardware to participants; and
- set-up and support for the computer software.

All coordinators and volunteers completed and returned the requested data. Expenses were taken as reported or estimated from external sources as necessary. Hardware and software costs were appropriately apportioned to groups. For example, the hardware costs reflected the number of computers that had to be loaned per group, while software costs reflected a share of the server and technical support costs. Time inputs were valued by multiplying them with appropriate unit costs (see S3 Supporting Information in S1 File). Coordinator time was valued at £30 per hour, volunteer time at £6.86 per hour and researcher time at £66 per hour. All costs were standardised at 2017/18 price levels and discounting was unnecessary because all costs related to a period of less than one year. Costs are presented in British pounds sterling (£), where £1 is equivalent to approximately 1.466 United States dollars (USD) based on 2017 purchasing power parity rates [65]

Costs for the various components were then summed to generate the following summary totals:

- Total cost per group and the average of this across groups (including and excluding hardware since more equipment loaning than anticipated became necessary to ensure participants had requisite standard computer hardware and it is unclear to what extent this would be replicated elsewhere)
- Average cost per participant per group, accounting for actual participant numbers observed for each group, and the average of this across groups (exc. hardware)
- Average cost per scheduled online attendance per group, accounting for actual total attendance observed for each group, and the average of this across group (exc. hardware)

## Results

### RQ1 feasibility results

#### Recruitment and retention (see Fig 1)

Thirty four people with aphasia were recruited over a period of 13 weeks, out of 67 screened. The consent rate of those eligible to take part was 72.34% (34/47). Twenty-two participants were recruited through the four community aphasia groups led by the co-ordinators. Three were recruited via self-referral and nine participants were referred via other community groups.

**Fig 1:**
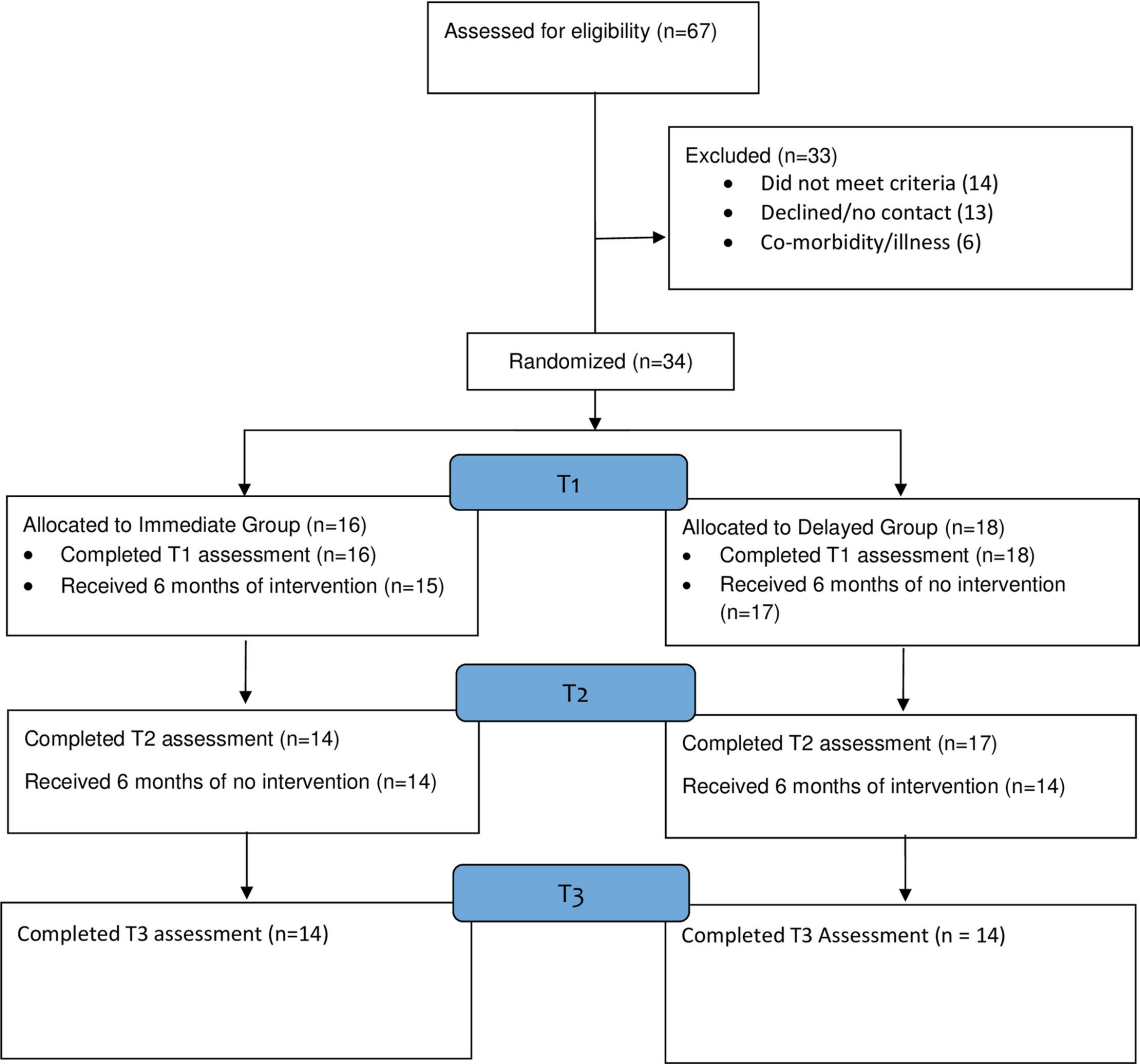
Study flow diagram.

Of those recruited, 31 began the intervention (91.18%). Twenty nine (85.3%) completed intervention and 27 (79.4%) completed all assessments. Reasons for attrition were family/health reasons (2); participant obtained employment (1); travel abroad (1); participant recruited by another research project which forbad involvement in this study (2); participant opted to withdraw (1). The total time from the first recruitment to the final T3 assessment was 18 months. The trial ended when data collection (minus attrition) was complete.

Participant characteristics are reported in Table 1. Data for age and months post stroke were not normally distributed. The conditions differed on two baseline characteristics. The delayed condition was older than the immediate condition (Mann-Whitney U = 77, p = .021) and contained more women (chi square (1) = 5.78, p = .016). The conditions did not differ with respect to months post stroke, presence of hemiplegia, educational attainment or the number who were in professional employment pre-stroke.

**Table 1:** Participant baseline characteristics.

	Immediate (n = 16)	Delayed (n = 18)	Total (n = 34)	Between Group Difference (Immediate vs Delayed)
Age Median (IQR)	51 (46.5–57.5)	65 (51.5–71.25)	53.5 (48.75–71)	P = .021
Months Post Stroke Median (IQR)	48 (29.75–85.25)	26.5 (11.75–79)	46.5 (15–83.75)	P = .33
Gender (number (%) female/ male)	4 (25%) / 16 (75%)	13 (72.2%) / 5 (27.8%)	17 (50%) / 17 (50%)	P = .016
Hemiplegia (number (%) with hemiplegia)	11 (68.7%)	13 (72.2%)	24 (70.6%)	P = .82
Education (number (%) who received higher education)	6 (37.5%)	4 (22.2%)	10 (29.4%)	P = .33
Employment (number (%) in professional/ non professional employment)	10 (62.5%) / 6 (37.5%)	7 (38.9%) / 11 (61.1%)	17 (50%) / 17 (50%)	P = .17

Seven participants failed to complete intervention and/or all assessments (see Fig 1). These participants did not differ from those with full data on any baseline characteristics (age, p = .6; months post stroke, p = .91; gender, p = .4; hemiplegia, p = .6; education, p = .8; employment, p = .4); although it should be acknowledged that the power to detect a difference was low. Two participants experienced adverse events during the trial, both of whom were in the delayed condition. These events were a hospital admission and referral to GP for depression.

#### Compliance with intervention

All four groups ran the 14 group sessions according to the planned schedule. The mean number of sessions attended per participant was 11.42 (s.d. 2.85), which is 81.6% of the intended dose. The median was 12 (IQR 11–13), and the range 3–14. Seven participants (22.6% of those who began intervention) missed no sessions. Fourteen (45.2%) missed one or two sessions. Ten (32.2%) missed 3 or more sessions. These ten included two participants who withdrew from the study after 3 and 4 sessions respectively, one because he did not like groups and one for family/health reasons. Aside from withdrawal, reasons for missed sessions were ill health, family events and failed internet connections.

The median number of times that participants logged into EVA Park outside the scheduled group sessions was 9, (IQR 4–13), range 0–81. The median amount of time spent in EVA Park, for group and independent access, was 27.03 hours (IQR 20.92–37.7), range 6.4–77.52 hours.

#### Fidelity

Inter-rater reliability of the fidelity checking scores was excellent. One hundred and twenty treatment components were double coded across five videos, with 96.7% agreement, Kappa = .92, p < .001.

Across all 18 videos that were evaluated, the mean fidelity score per treatment component was 1.76 (/2; range 1.45–2; median 1.82). In these videos 81.9% of the applicable treatment components were fully present (scoring 2), 12.6% were present to some degree (scoring 1) and 5.5% were absent (scoring 0). Ten videos were taken from the first 5 sessions of intervention, and 8 from the last 5 sessions. Fidelity scores did not differ for early and late sessions (early mean score = 1.758; late mean score = 1.761; t (16) = -.039, p = .97). Videos were also sampled from each intervention group. Their scores are reported in Table 2 and were not significantly different (Kruskal-Wallis H (3) = 5.16, p = .16).

**Table 2:** Mean treatment fidelity scores across intervention groups.

Intervention Group	Number of Videos Scored	Mean Fidelity Score (s.d)
North	6	1.64 (.17)
South	5	1.86 (.09)
East	3	1.78 (.18)
West	4	1.79 (.19)

### RQ2 outcome measures

Scores on all outcome measures at the three time points are reported in Table 3. The primary ANCOVA analysis compared scores at T2 with scores at T1 as the covariate. This was first performed on an ITT basis. Therefore, three T2 scores were imputed (two in the immediate condition and one in the delayed condition), by carrying forward the last data point. T3 data is reported on a Per-Protocol basis. It includes all participants who completed the intervention and the T3 assessments.

**Table 3:** Mean (sd) *[Median; IQR]* scores on the outcome measures at T1, T2 and T3; treatment effects in the primary analysis.

	T1		T2		T3		Between Group Difference at T2		
	Immediate Mean (sd) *[Median; IQR]* (n = 16)	Delayed Mean (sd) *[Median; IQR]* (n = 18)	Immediate Mean (sd) (n = 16)	Delayed Mean (sd) (n = 18)	Immediate Mean (sd) (n = 14)	Delayed Mean (sd) (n = 14)	ANCOVA F (df1, df2)	p value	η_p_^2^
WEMWBS	53.00 (9.20)	46.83 (10.75)	54.69 (12.30)	49.94 (9.99)	53.86 (9.34)	52.86 (14.49)	.06 (1, 31)	.81	.002
CADL-2	89.37 (7.02) *[92*.*0; 85–93*.*75]*	81.28 (8.37) *[83*.*5; 76–88]*	89.81 (7.61)	83.00 (8.49)	89.21 (6.87)	87.29 (7.72)	.55 (1,31)	.46	.018
SCS	83.87 (17.20)	81.22 (17.77)	88.12 (17.04)	85.00 (17.16)	89.43 (15.03)	86.65 (16.76)	.09 (1, 31)	.77	.003
WAB-R	78.22 (13.19)	70.48 (14.64)	81.86 (12.57)	71.79 (12.27)	79.99 (13.29)	77.96 (11.19)	4.11 (1, 31)	.051	.12
SAQOL-39g	3.78 (.57)	3.31 (.66)	3.73 (.72)	3.35 (.65)	3.78 (.72)	3.37 (.78)	.13 (1, 31)	.72	.004

WEMWBS: Warwick Edinburgh Mental Wellbeing Scale; CADL-2: Communication Activities of Daily Living -2; SCS: Social Connectedness Scale; WAB-R: Western Aphasia Battery—Revised; SAQOL-39g: Stroke and Aphasia Quality of Life-39 generic version

Preliminary checks were conducted on the T1 and T2 data to ensure that there was no violation of the assumptions of normality, linearity, homogeneity of variance, homogeneity of regression slopes, and reliable measurement of the covariate. These assumptions were met with all the data except CADL-2 scores at T1, where data in both conditions were not normally distributed (Shapiro Wilk p < .05). However, as the skewness value was only just outside the acceptable range (skewness = -1.11) the ANCOVA was conducted.

After adjusting for T1 scores, there were no significant differences between the immediate and delayed conditions at T2. The only measure that approached significance was the WAB-R (p = .051) with a medium effect size (partial eta squared = .12). For all measures there was a strong relationship between T1 and T2 scores (partial eta squared range = .55 - .82). The ANCOVA analyses were repeated on a Per Protocol basis (immediate n = 14; delayed n = 17), with no difference in the findings. The WAB-R result was again just short of significance (p = .051; partial eta squared = .13). Therefore, both the ITT and Per-Protocol ANCOVA analyses failed to detect any indicative effects of therapy on these measures.

Table 4 reports pre and post intervention scores on all measures. Scores are reported for all participants who completed the relevant assessment points and the intervention. For participants in the immediate condition pre/post scores are drawn from T1 and T2; while for those in the delayed condition they are drawn from T2 and T3. All data were normally distributed except for the pre intervention CADL-2 data (Shapiro Wilk, p < .05).

**Table 4:** Mean (sd) *[Median; IRQ]* scores for all participants on the outcome measures pre and post intervention; n = 28.

	Pre Intervention	Post Intervention	Significance	Effect Size
WEMWBS	52.52 (9.67)	54.32 (13.46)	t (27) = 1.06, p = 0.30	*d* = .24
CADL-2	86.82, (8.22) *[89; 81*.*25–93]*	88.32 (7.81) *[88*.*5; 81*.*75–95*.*75]*	Z = -.97, p = 0.33	r = .13
SCS	86.57 (15.69)	89.14 (15.70)	t (27) = 1.07, p = 0.29	*d* = .20
WAB-R	75.17 (13.34)	79.97 (12.27)	t (27) = 3.05, p = .005	*d* = .58
SAQOL-39g	3.61 (.65)	3.56 (.77)	t (27) = -.81, p = .42	*d* = -.15

WEMWBS: Warwick Edinburgh Mental Wellbeing Scale; CADL-2: Communication Activities of Daily Living -2; SCS: Social Connectedness Scale; WAB-R: Western Aphasia Battery-Revised; SAQOL-39g: Stroke and Aphasia Quality of Life-39 generic version

Change on most measures was non-significant, and with small effect sizes. Only the WAB-R showed a significant improvement, with a medium effect size.

### RQ3 cost estimates

Cost estimates are summarised in Table 5, with further details of each component described in S4 and S5 Supporting Information in S1 File. Total costs per group ranged between £6,516 and £11,316 ($9,552 USD and $16,589 USD) excluding hardware, and £7,483 and £12,562 ($10,970 USD and $18,416 USD) including hardware. The most costly component across all groups was the training for coordinators and volunteers at an average cost of £3,414 ($5,001 USD) per group.

**Table 5:** Summary of costs.

	Total cost (£, 2017–18 prices)				
	North	East	South	West	Mean
Total cost for training	£4,627	£1,738	£3,826	£3,465	£3,414
Total cost for project manager inputs to groups	£1,835	£858	£1,416	£1,027	£1,284
Total cost for coordinator inputs to groups	£2,610	£1,470	£1,829	£2,220	£2,032
Total cost for volunteer inputs to groups	£829	£1,034	£779	£1,018	£915
Total hardware costs	£1,245	£967	£697	£692	£901
Total software costs	£1,416	£1,416	£1,416	£1,416	£1,416
**TOTAL COST FOR GROUP (inc. hardware)**	**£12,562**	**£7,483**	**£9,963**	**£9,838**	**£9,961**
**TOTAL COST FOR GROUP (exc. hardware)**	**£11,316**	**£6,516**	**£9,265**	**£9,146**	**£9,061**
Average cost per participant (exc. hardware)	£2,263	£724	£1,324	£1,143	£1,364
Average cost per online attendance (exc. hardware)	£195	£68	£101	£91	£114

(£1 is equivalent to approximately 1.466 United States dollars (USD) based on 2017 purchasing power parity rates)

The different implementation contexts across the groups were reflected in the costs. For example, one group (East) required considerably less researcher time for the training compared with the other three because its training sessions took place at the project managers’ university base and thus incurred no travel time or expenses for the trainers (whose time was more expensive than that of coordinators and volunteers).

The average cost per participant across the four groups was £1,364 ($2,000 USD) (excluding hardware). This was naturally driven by group size as much as the input variations across the groups. The group with the lowest input costs had the largest group size so generated an average cost per participant of £724 ($1,061 USD) as compared with averages ranging between £1,143 and £2,263 ($1,676 USD and $3,318 USD) for the other three groups. Conversely, the group with the largest input costs also had the smallest group size, thus generating a much larger average cost per participant of £2,263 ($3,318 USD).

## Discussion

The first research question addressed by this study was whether delivery of group social support to people with aphasia via EVA Park was feasible. Results showed that recruitment targets were met, and full data collection was achieved with just under 80% of all participants. All four intervention groups delivered the intervention as planned, and participants, on average, attended over 80% of sessions. Usage data indicated that all bar one of the participants visited EVA Park outside scheduled sessions, with a median of 9 unscheduled log ins per participant. This demonstrated a level of involvement in the platform and possible compliance with the challenge tasks that were set between sessions. Fidelity checking indicated that there was minimal drift from the intervention as described in the manual, despite the fact that delivery was delegated to community co-ordinators and volunteers. Fidelity was maintained across the treatment period, with both early and late sessions scoring highly.

It is difficult to compare our feasibility results with those of previous studies, given the variation in methods. However, the percentage of consents to those screened compares well with some trials of face-to-face aphasia therapy. For example, in the ACT NoW study 170 participants were randomised from 2074 screened [66] and a more recent trial of intensive aphasia therapy, recruited 24.7% of those screened [67]. The fact that we recruited 34 participants from 67 screens (50.7%) suggests that people with aphasia are willing to take part in studies of remote intervention. This was similarly the case in Pitt et al [36] where over 67% of those screened progressed to intervention. Our participants were, on average, younger than typical stroke survivors [68]. Just under a third (29.4%) had also attended higher education, which is a higher proportion than the UK average for their age range [69]. Recruitment across a wider and more typical stroke population might be more challenging.

Previous studies of group interventions have reported both less attrition than we experienced ([12]: 17.2%) and more ([16]: 26.3%). In our study, participants’ involvement extended over a year, making attrition more likely than in studies of a shorter duration. Participants’ compliance with the treatment regime is not always reported in aphasia intervention studies, but some papers record higher rates of attendance than were achieved here [15, 70]. These studies delivered intervention over a shorter period, in one case very intensively, which may reduce the risk of missed sessions. Some individuals missed sessions because of problems with the technology and a loss of internet connectivity. Similar difficulties are reported by Pitt et al [36] in their study of remote group therapy. This suggests that more robust technologies and improved internet connections would improve compliance with remote and VR therapies. Conversely, one potential risk for attendance was eliminated by our remote delivery format, namely transport difficulties. This was underscored for those in the immediate condition who received intervention during a period of heavy snow, when EVA Park groups could progress as planned, despite several members being house bound. People with aphasia express complex reasons behind the decision to attend (or not attend) community support groups [29]. Our qualitative data may show similarly complex issues behind our attrition and attendance data.

Treatment fidelity is often neglected in aphasia intervention research. A review of 149 aphasia treatment papers conducted between 2002 and 2011, found that only 14% reported on treatment fidelity [71]. In a more recent review, covering studies that were published between 2012 and 2017, explicit reporting on treatment fidelity was still only present in 21% of studies [72]. When fidelity is assessed, review of videotaped sessions, checked for adherence to the treatment manual, is a commonly used method [71]. We employed this approach and our findings suggest that adherence to the core components of a remote group intervention can be maintained.

The second research question asked whether a programme of group social support delivered in EVA Park would improve scores on measures of well-being, communication, social connectedness, language and quality of life. No measures showed change in the primary analysis comparing the immediate and delayed groups at T2. In the secondary analysis, comparing pre to post intervention scores across all participants, only the Western Aphasia Battery-Revised showed evidence of improvement. Accounting for this one result is difficult. The WAB-R was not a primary outcome measure, and although the group sessions stimulated language, the intervention placed a greater emphasis on psychosocial and communicative factors. Given the number of tests administered, the WAB-R result may have been a type one error. However, even if alpha is adjusted to .01, with a Bonferroni correction for five comparisons, significance is still achieved (on the WAB-R result p < .005). It is important to note that this was not a controlled analysis. Results cannot, therefore, be unambiguously attributed to the treatment. Analyses of all other measures were statistically non significant with small effect sizes.

Drawing conclusions about the effectiveness of the intervention from this trial would be premature, given that this was a feasibility study with low power. However, other small scale studies of group intervention have shown change, both in controlled [12] and uncontrolled [16] conditions. The most direct comparison would be with a previous study of remote group intervention, where 19 participants improved significantly on the Assessment of Living with Aphasia, the Quality of Communication Life Scale and the Comprehensive Aphasia Test [36]. There were several differences between this and the Pitt et al study. While the amount of intervention was similar (18 vs 21 hours) in Pitt et al it was delivered weekly rather than every two weeks. Their therapy groups were also smaller, with no more than four participants. This may have impacted on individual engagement, and on the opportunities for communication provided to each participant. In Pitt et al, the therapy was administered by a qualified therapist, rather than delegated to community co-ordinators and volunteers. Pitt et al also employed different measures, which may have been more sensitive to treatment effects.

Our third research question focused on the cost of the intervention. The average cost of implementing each group was £9,061 ($13,283 USD), with a range of £6,516 to £11,316 ($9,552 USD to $16,589 USD) (all values are excluding hardware). Two factors seemed to drive this range. One was the distance between the stroke service and the research team base, resulting in high project manager travel costs particularly for delivering training. The other was the relative amount of time given by volunteers. Thus, the group with the highest volunteer involvement was also the cheapest, as this reduced the input needed from the more expensive co-ordinator and project managers. The average per participant cost for the full episode of intervention was £1,364 ($2,000 USD), with a range of £724 to £2,263 ($1,061 USD to $3,318 USD). This range partly reflected the discrepant group costs, but was also a consequence of different group sizes.

One group in particular incurred substantially greater travel expenses for the project managers due to distance. In a post-hoc scenario analysis, we substituted some of these high values with lower ones to gauge the impact on total costs. First, for the three groups requiring the greatest trainer time because trainers travelled to them, we instead substituted the trainer time cost (£1,265; $1,854 USD) of the group with the lowest such cost. Second, for the group with the highest researcher travel expenses for training (because it was the furthest site), we substituted the average travel expenses for the other three groups (£124; $182 USD). Together, these two alternative scenarios resulted in average total costs per group falling from £9,061 to £7,824 ($13,283 USD to $11,470 USD).

The cost data has some useful implications for models of implementation by potential service providers and in a more definitive trial. All costs were inflated by the need for hardware provision. Services might therefore be faced with a difficult choice between restricting this provision to those who already have computers, or adopting the more inclusive, but expensive, option of including those who need hardware loans. Training reflected a significant portion of the costs. Services might therefore seek to maximise their investment in training, for example by training teams that deliver multiple groups over time. Exploring cheaper models for training, for example by supplementing face-to-face delivery with online resources, might also be an option. Finally, services need to make efforts to secure and maintain a high level of volunteer engagement to help contain costs.

There are limitations in the cost data. It is difficult to know how this provision compares to more conventional modes of delivery. Wenke et al [73] assessed the costs of embedding different service delivery models for aphasia treatment in three sub-acute Australian facilities. Their per participant costs ranged from AUD3289 (for group therapy) to AUD6655 (for therapy delivered by a SLT assistant), which under current conversion rates equate to £1806 and £3655. However, their dose (between 84–91 hours) was different from ours, as was the treatment context. Cost analysis of face-to-face community-based group provision for people with aphasia has previously been undertaken [74]. However, comparisons with our study are problematic as the data was collected 17 years ago (2002) and relate to different models of intervention from that tested in our study, for example with respect to dose and staffing. A further limitation is that our data are purely descriptive and only consider costs related to the intervention. Assessing the value of the intervention requires methods that assess costs more comprehensively (including overall care costs for participants) against benefit.

### Future implications

Our findings indicate that it would be feasible to conduct a larger trial of remote group intervention for people with aphasia in EVA Park. However, the lack of any indicative treatment effects found in this study suggest that revisions should be made to the intervention and assessment of outcomes. Here, findings from our qualitative data will be crucial, but possible amendments include the use of a more intensive regime and smaller intervention groups. Other programmes of group social support have also involved family members [15], which might be considered for a future trial. Preliminary inspection of our qualitative findings has unearthed quite widespread difficulties with internet connectivity and sound quality, which need to be addressed before progression to a larger trial. Turning to assessment, the outcome measures should be reviewed, and possibly restricted to tools that were designed specifically for people with aphasia. The Assessment of Living with Aphasia might be considered as a primary outcome measure, given that this has shown treatment effects in a comparable study [36]. Our economic data can inform decisions about which components of the intervention are worth focussing on for a reliable assessment of costs. This should form part of a comprehensive economic evaluation, incorporating overall care costs for participants and relevant outcomes.

## Supporting information

S1 Checklist CONSORT 2010 checklist of information to include when reporting a randomised trial*. (DOC)

S1 File (DOCX)

S1 Protocol (DOCX)

S1 Dataset (SAV)

## References

[1] Northcott S, Marshall J, Hilari K. What factors predict who will have a strong social network following a stroke? Journal of Speech Language and Hearing Research, 2016; 59: 772–83. 10.1044/2016_JSLHR-L-15-0201 27401538

[2] Cruice M, Worrall L, Hickson L. Quantifying aphasic people's social lives in the context of non-aphasic peers. Aphasiology, 2006; 20: 1210–1225. 10.1080/02687030600790136

[3] Hilari K, Wiggins R, Roy P, Byng S, Smith S. Predictors of health-related quality of life (HRQL) in people with chronic aphasia. Aphasiology, 2010; 17: 365–381. 10.1080/02687030244000725

[4] Kauhanen M, Korpelainen J, Hiltunen P, Määttä R, Mononen H, Brusin E, et al Aphasia, depression, and non-verbal cognitive impairment in ischaemic stroke. Cerebrovascular Diseases, 2000; 10: 455–61. 10.1159/000016107 11070376

[5] Shadden B. Aphasia as identity theft: Theory and practice, Aphasiology, 2005; 19: 211–223, 10.1080/02687930444000697

[6] Brown K, Worrall LE, Davidson B, Howe T. Living successfully with aphasia: a qualitative meta-analysis of the perspectives of individuals with aphasia, family members, and speech-language pathologists. International Journal of Speech and Language Pathology, 2012; 14: 141–155 10.3109/17549507.2011.632026 22149648

[7] Simmons-Mackie N, Elman R. Negotiation of identity in group therapy for aphasia: the Aphasia Café, International Journal of Language & Communication Disorders, 2011; 46(3): 312–323. 10.3109/13682822.2010.507616Elman 1999 21575072

[8] Sherratt S, Simmons-Mackie N. Shared humour in aphasia groups: “They should be called cheer groups”, Aphasiology, 2016; 30: 1039–1057, 10.1080/02687038.2015.1092495

[9] Vickers C. Social networks after the onset of aphasia: The impact of aphasia group attendance. Aphasiology, 2010; 24: 902–913.

[10] Shadden B. Rebuilding identity through stroke support groups: Embracing the person with aphasia and significant others. In Elman R. (ed) Group treatment of neurogenic communication disorders: The expert clinician’s approach (2nd edition), 2007; 111–126. San Diego CA: Plural.

[11] Lanyon L, Rose M, Worrall L. The efficacy of outpatient and community-based aphasia group interventions: A systematic review, International Journal of Speech-Language Pathology, 2013; 15: 359–374, 10.3109/17549507.2012.752865 23336826

[12] Elman R, Bernstein Ellis E. The Efficacy of Group Communication Treatment in Adults With Chronic Aphasia. Journal of Speech, Language, and Hearing Research, 1999; 42: 411–419. 10.1044/jslhr.4202.411 10229456

[13] Pulvermüller F, Neininger B, Elbert T, Mohr B, Rockstroh B, Koebbel P, et al Constraint-induced therapy of chronic aphasia after stroke. Stroke, 2001; 32: 1621–1626. 10.1161/01.str.32.7.1621 11441210

[14] Ross A, Winslow I, Marchant P, Brumfitt S. Evaluation of communication, life participation and psychological well‐being in chronic aphasia: The influence of group intervention, Aphasiology, 2006; 20: 427–448, 10.1080/02687030500532786

[15] Attard M, Loupis Y, Togher L, Rose M. The efficacy of an inter-disciplinary community aphasia group for living well with aphasia, Aphasiology, 2018; 32: 105–138, 10.1080/02687038.2017.1381877

[16] van der Gaag A, Smith L, Davis S, Moss B, Cornelius V, Laing S, et al Therapy and support services for people with long-term stroke and aphasia and their relatives: a six-month follow-up study. Clinical Rehabilitation, 2005; 19: 372–380. 10.1191/0269215505cr785oa 15929505

[17] Corsten S, Konradi J, Schimpf E, Hardering F, Keilmann A, Improving quality of life in aphasia—Evidence for the effectiveness of the biographic-narrative approach, Aphasiology, 2014; 28: 440–452, 10.1080/02687038.2013.843154

[18] Hartke R, King R, Denby F. The Use of Writing Groups to Facilitate Adaptation After Stroke, Topics in Stroke Rehabilitation, 2007; 14: 26–37, 10.1310/tsr1401-26 17311788

[19] Attard M, Loupis Y, Togher L, Rose M. Experiences of people with severe aphasia and spouses attending an Interdisciplinary Community Aphasia Group, Disability and Rehabilitation, 2019; 10.1080/09638288.2018.1526336 30652928

[20] Corsten A, Schimpf E, Konradi J, Keilmann A, Hardering F. The participants’ perspective: how biographic-narrative intervention influences identity negotiation and quality of life. International Journal of Language and Communication Disorders, 2015; 50: 788–800. 10.1111/1460-6984.12173 26123497

[21] Power E, Thomas E, Worrall L, et al Development and validation of Australian aphasia rehabilitation best practice statements using the RAND/UCLA appropriateness method. BMJ Open 2015; 5:e007641 10.1136/bmjopen-2015-007641 26137883 PMC4499686

[22] Verna A, Davidson B, Rose T. Speech-language pathology services for people with aphasia: A survey of current practice in Australia, International Journal of Speech-Language Pathology, 2009; 11: 191–205, 10.1080/17549500902726059

[23] Rose M, Attard M. Practices and challenges in community aphasia groups in Australia: Results of a national survey, International Journal of Speech-Language Pathology, 2015; 17: 241–251, 10.3109/17549507.2015.1010582 25739326

[24] Dorning H, Davies M, Ariti C, Allen K, Georghiou T. Knowing you’re not alone: Understanding peer support for stroke survivors 2016; The Nuffield Trust, 2016.

[25] Naylor C, Mundle C, Weaks L, Buck D. Volunteering in health and care: Securing a sustainable future. 2008London: The King’s Fund; 2008

[26] Farrell C, Bryant W. Voluntary work for adults with mental health problems: a route to inclusion? A review of the literature. British Journal of Occupational Therapy. 2008; 72: 163–73.

[27] Geddes J, Chamberlain M. Improving social outcome after stroke: an evaluation of the volunteer stroke scheme. Clinical Rehabilitation. 1994; 8:116–126.

[28] Marshall J, Sacchett C. Does the Volunteer Stroke Scheme improve social outcome after stroke? A response to Geddes and Chamberlain. Clinical Rehabilitation. 1996; 10:104–109.

[29] Lanyon L, Worrall L, Rose M. “It’s not really worth my while”: understanding contextual factors contributing to decisions to participate in community aphasia groups, Disability and Rehabilitation, 2019; 41: 1024–1036, 10.1080/09638288.2017.1419290 29320876

[30] Palsbo S. Equivalence of functional communication assessment in speech pathology using videoconferencing. Journal of Telemedicine and Telecare, 2007; 13: 40–43. 10.1258/135763307779701121 17288658

[31] Hill A, Theodoros D, Russell T, Ward E, Wootton R. The effects of aphasia severity on the ability to assess language disorders via telerehabilitation. Aphasiology, 2009; 23: 627–642.

[32] Choi Y. Park H, Paik N. A telerehabilitation approach for chronic aphasia following stroke. Telemedicine and e-Health, 2016; 22: 434–440. 10.1089/tmj.2015.0138 26544535

[33] Hall N, Boisvert M, Steele R. Telepractice in the assessment and treatment of individuals with aphasia: A systematic review. International Journal of Telerehabilitation, 2013; 5: 27 10.5195/ijt.2013.6119 25945211 PMC4296832

[34] Woolf C, Caute A, Haigh Z, Galliers J, Wilson S, Kessie A, et al A comparison of remote therapy, face to face therapy and an attention control intervention for people with aphasia: A quasi-randomised controlled feasibility study. Clinical rehabilitation, 2016; 30: 359–373 10.1177/0269215515582074 25911523

[35] Pitt R, Theodoros D, Hill A, Russell T. The development and feasibility of an online aphasia group intervention and networking program–TeleGAIN, International Journal of Speech-Language Pathology, 2017; 10.1080/17549507.2017.1369567 28868932

[36] Pitt R, Theodoros D, Hill A, Russell T. The impact of the telerehabilitation group aphasia intervention and networking programme on communication, participation, and quality of life in people with aphasia, International Journal of Speech-Language Pathology, 2018; 10.1080/17549507.2018.1488990 30200788

[37] Pitt R, Hill A, Theodoros D, Russell T. “I definitely think it’s a feasible and worthwhile option”: perspectives of speech-language pathologists providing online aphasia group therapy, Aphasiology, 2018; 32: 1031–1053, 10.1080/02687038.2018.1482403

[38] Wilson S, Roper A, Marshall J, Galliers J, Devane N, Booth T, et al Codesign for people with aphasia through tangible design languages. CoDesign: International Journal of CoCreation in Design and the Arts, 2015; 10.1080/15710882.2014.997744

[39] Marshall J, Booth T, Devane N, Galliers J, Greenwood H, Hilari K, et al Evaluating the Benefits of Aphasia Intervention Delivered in Virtual Reality: Results of a Quasi-Randomised Study. PLoS ONE 2016; 11(8): e0160381 10.1371/journal.pone.0160381 27518188 PMC4982664

[40] Amaya A, Woolf C, Devane N, Galliers J, Talbot T, Wilson S, et al Receiving Aphasia Intervention in a Virtual Environment: The Participants’ Perspective. Aphasiology, 2018; 10.1080/02687038.2018.1431831

[41] Galliers J, Wilson S, Marshall J, Talbot R, Devane N, Booth T, et al Experiencing EVA Park, a Multi-User Virtual World for People with Aphasia. ACM Transactions on Accessible Computing 2017; 10, 4, Article 15 (October 2017), 24 pages. 10.1145/3075300 29270243 PMC5736157

[42] Marshall J, Devane N, Edmonds L, Talbot R, Wilson S, Woolf C, et al Delivering word retrieval therapies for people with aphasia in a virtual communication environment. Aphasiology, 2018; 10.1080/02687038.2018.1488237

[43] Carragher M, Talbot R, Devane N, Rose M, Marshall J. Delivering storytelling intervention in the virtual world of EVA Park, Aphasiology, 2018; 32: sup1, 37–39, 10.1080/02687038.2018.1484880

[44] Pearl G, Cruice M. Facilitating the involvement of people with aphasia in stroke research by developing communicatively accessible research resources. Topics in Language Disorders, 2017; 37: 67–84.

[45] Enderby P, Crow E. Frenchay aphasia screening test: validity and comparability. Disability and Rehabilitation, 1996; 18: 238–240. 10.3109/09638289609166307 8743301

[46] Caute A, Woolf C, Wilson S, Stokes C, Monnelly K, Cruice M, et al Technology-Enhanced Reading Therapy for People With Aphasia: Findings From a Quasirandomized Waitlist Controlled Study. Journal of Speech, Language, and Hearing Research, 2020 10.1044/2019_JSLHR-L-18-0484 31765277

[47] Caute A., Galliers J., Marshall J., Monnelly K., Woolf C. The development of a novel Dynamic Assessment of Computer Learning; in preparation

[48] Julious SA. Sample size of 12 per group rule of thumb for a pilot study. Pharmaceutical Statistics. 2005; 4: 287–291.

[49] Sim J, Lewis M. The size of a pilot study for a clinical trial should be calculated in relation to precision and efficiency. Journal of Clinical Epidemiology, 2012; 65: 301–308. 10.1016/j.jclinepi.2011.07.011 22169081

[50] Attard M, Lanyon L, Togher L, Rose M. Consumer perspectives on community aphasia groups: a narrative literature review in the context of psychological well-being. Aphasiology, 2015; 29: 983–1019; 10.1080/02687038.2015.1016888

[51] Shadden B, Agan J. Renegotiation of identity: The social context of aphasia support groups. Topics in Language Disorders, 2004; 24: 174–186.

[52] Holland A. Counselling in communication disorders: A wellness perspective. 2007; San Diego, CA: Plural.

[53] Pound C, Parr S, Lindsay J, Woolf C, Beyond Aphasia, 2000; Telford: Winslow Press.

[54] Rippon S, Hopkins T, Head, hands and heart: asset-based approaches in health care A review of the conceptual evidence and case studies of asset-based approaches in health, care and wellbeing. The Health Foundation, 2015; www.health.org.uk downloaded 15.02.17

[55] Seligman M, Steen T, Park N, Peterson C. Positive psychology in progress. Empirical validation of interventions. American Psychologist, 2005; 60: 410–421. 10.1037/0003-066X.60.5.410 16045394

[56] Shiggins C, Soskolne V, Olenik D, Pearl G, Haaland-Johnanse L, Isaksen J, et al Towards an asset-based approach to promoting and sustaining well-being for people with aphasia and their families: an international exploratory study. Aphasiology, 2018; 10.1080/02687038.2018.1548690

[57] Tennant R, Hiller L, Fishwick R, Platt S, Joseph S, Weich S, et al The Warwick-Edinburgh mental well-being scale (WEMWBS): development and UK validation. Health and Quality of Life Outcomes, 2007; 5(1): 63. 18042300 10.1186/1477-7525-5-63 PMC2222612

[58] Holland A, Frattali C, Fromm D. Communication Activities of Daily Living-2. 1999; Austen TX: Pro-Ed

[59] Lee R, Robbins S, Measuring belongingness: The Social Connectedness and the Social Assurance Scales. Journal of Counselling Psychology, 1995; 42: 232–241

[60] Kertesz A, Western Aphasia Battery-Revised. 2006; Pearson.

[61] Hilari K, Lamping D, Smith S, Northcott S, Lamb A, & Marshall J. Psychometric properties of the Stroke and Aphasia Quality of Life Scale (SAQOL-39) in a generic stroke population. Clinical Rehabilitation, 2009; 23: 544–557. 10.1177/0269215508101729 19447841

[62] Brady MC, Kelly H, Godwin J, Enderby P, Campbell P. Speech and language therapy for aphasia following stroke. Cochrane Database of Systematic Reviews 2016; Issue 6: Art. No.: CD000425. 10.1002/14651858.CD000425.pub4 27245310 PMC8078645

[63] Wallace S, Worrall L, Rose T, Le Dorze G, Breitenstein C, Hilari K, et al A core outcome set for aphasia treatment research: the ROMA consensus statement. International Journal of Stroke, 2018; 180–185. 10.1177/1747493018806200 30303810

[64] Hilari K, Behn N, Marshall J, Simpson A, Thomas S, Northcott S, et al Adjustment with aphasia after stroke: study protocol for a pilot feasibility randomised controlled trial for Supporting wellbeing through PEeR Befriending (SUPERB). Pilot and Feasibility Studies, 2019; 5, 14 10.1186/s40814-019-0397-6 30693094 PMC6341752

[65] Organisation for Economic Co-operation and Development. Purchasing power parities, comparative price levels. https://data.oecd.org/conversion/purchasing-power-parities-ppp.htm. (Accessed 21 May 2019)

[66] Bowen A, Hesketh A, Patchick A, Young A, Davies L, Vail A, et al Effectiveness of enhanced communication therapy in the first four months after stroke for aphasia. British Medical Journal, 2012; 345, 7868, 10.1136/bmj.e4407 22797843 PMC3396461

[67] Breitenstein C, Grewe T, Flöel A, et al Intensive speech and language therapy in patients with chronic aphasia after stroke: a randomised, open-label, blinded-endpoint, controlled trial in a health-care setting. Lancet, 2017; 389: 1528–38 10.1016/S0140-6736(17)30067-3 28256356

[68] Lee S, Shafe ACE, Cowie MR. UK stroke incidence, mortality and cardiovascular risk management 1999–2008: time-trend analysis from the General Practice Research Database. BMJ Open 2011;1:e000269 10.1136/bmjopen-2011-000269 22021893 PMC3211058

[69] Bolton P. (2012) Education: Historical statistics; Standard Note: SN/SG/4252. House of Commons Library. https://researchbriefings.files.parliament.uk/documents/SN04252/SN04252.pdf. Accessed on 31st May, 2019

[70] Rodriguez A, Worrall L, Brown K, Grohn B, McKinnon E, Pearson C, et al Aphasia LIFT: Exploratory investigation of an intensive comprehensive aphasia programme, Aphasiology, 2013; 27:11, 1339–1361, 10.1080/02687038.2013.825759

[71] Hinckley J, Douglas N. Treatment fidelity: Its importance and reported frequency in aphasia treatment studies. American Journal of Speech-Language Pathology, 2013; 22: S279–S284. 10.1044/1058-0360(2012/12-0092)-0360(2012/12-0092) 23695904

[72] Brogan E, Ciccone N, Godecke E. Treatment fidelity in aphasia randomised controlled trials, Aphasiology, 2019; 33: 759–779, 10.1080/02687038.2019.1576442

[73] Wenke R, Lawrie M, Hobson T, Comben W, Romano M, Ward E, et al Feasibility and cost analysis of implementing high intensity aphasia clinics within a sub-acute setting. International Journal of Speech-Language Pathology 2014; 16(3): 250–259. 10.3109/17549507.2014.887777 24597463

[74] Van der Gaag A, Brooks R. Economic aspects of a therapy and support service for people with long-term stroke and aphasia. International Journal of Language and Communication Disorders, 2008; 43: 233–244. 10.1080/13682820701560376 18446573

