# Supplementary material for: A randomised trial of social support group intervention for people with aphasia: A Novel application of virtual reality
Source: PLoS One. 2020 Sep 24;15(9):e0239715. doi: 10.1371/journal.pone.0239715 (PMC7514104; doi:10.1371/journal.pone.0239715)
Supplement: S1 File — (DOCX) [file pone.0239715.s002.docx]

S1 Supporting Information: List of Intervention Sessions and Sample Session Plans

List of Sessions

1. EVA

2. YOU

3. APHASIA

4. MUSIC

5. RESILIENCE

6. COMEDY

7. PERSONAL STRENGTHS

8. INTRODUCE A PROJECT

9. ROLES

10. SPORTS

11. EATING OUT

12. ART

13. LITERATURE

14. REVIEW and PARTY

# SESSION 5

## RESILIENCE

### Outline:

All 8 members

1. Introduce the concept of resilience, what it means, and the idea that it can be developed

Split into 2 group of 4:

1. Explore risk taking and links to self-efficacy

## Aims:

- Members will understand qualities associated with resilience
- Member will identify risks they have taken and the impact on their self-concept

| Activity | Place | Content | Materials | Time |
| --- | --- | --- | --- | --- |
| Introduction  x8 | At a board behind health centre | What does resilience mean?  Becoming more resilient is possible, change and growth are possible | Qualities associated with resilience on board (appendix B) | 15 |
| Resilience skills  x4 | Campfire  (behind disco and Tiki island) | 1. Risk taking  Why did you sign up to this research?  What are your expectations?  What risks did you take just to be here?  Is taking risks necessary?  What does risk taking have to do with resilience?  Did you learn something about yourself by stepping off into the unknown?  (Did it show you your ability to cope/strength?)  Share a recent experience of taking risk e.g. taking part in research in a virtual world! |  | 40 |
| Activity  x4 | Pond | Diving |  | 10 |
| Discussion  x8 | Treehouse | Three good things |  | 20 |

Equipment and resources:

**Appendix B**: List of characteristics displayed by prisoners of war who did not develop post-traumatic stress disorder

- Optimism
- Altruism – helping others reduce stress
- Having an enduring set of beliefs or moral compass
- Faith and spirituality
- Humour
- Having a role model
- Having social supports
- Being able to leave ones comfort zone (facing fear / risk taking)
- Having a mission or meaning in life
- Having some training in mastering challenges

### Challenge tasks:

Meet up with one other member during the week in EVA Park. Find out their favourite TV comedy programme

# SESSION 6:

## COMEDY

### Outline:

All 8 members

1. Introduction to today’s session: We will discuss different types of comedy, what you like/dislike, benefits of comedy.

Feedback from challenge task: what did you find out about your partner’s favourite comedy?

1. Viewing comedy clips
   - 1. Mr Bean
     2. Laurel and Hardy
     3. Monty Python Dead Parrot

The group will watch the clip and discuss:

What was it?

What did you like about the clip?

Did it make you laugh (why/why not?)

1. Comedy in your life

Tell the group about a funny thing that happened to you or a friend or member of your family

How does comedy add to your life?

1. Prepare for challenge task

### Aims:

- enable self-expression
- identify common and divergent opinions
- identify sources of well-being

| Activity | Place | Content | Materials | Time |
| --- | --- | --- | --- | --- |
| Introduction | Café | Overview of session |  | 15 |
| Viewing Comedy clips | Media Screens: near the square, in the red house, in health centre reception, behind treehouse | Watch clips  *What is it?*  *Did it make you laugh?*  *What did you like about it?* | Video clips | 45 |
| Comedy in your life | Café | Tell a funny story from your life  How does comedy add to your life? Humour was identified as a characteristic that protected against PTSD |  | 15 |

### Equipment and resources:

Video clips

### Challenge tasks:

Prepare to share something that you like about yourself

Talk to someone who knows you well e.g. a friend or family member.  Ask them what they think your personal strengths are.

Arrange to meet another group member in EVA before the next session.  Tell them about something you have succeeded in recently.  This could be a big success, or something small that made you feel good.

S2 Supporting Information: Fidelity Checklist

|  | **Core components** | **Present/Yes** | ***Present to some degree*** | **Absent/No** | **N/A** | **Notes** |
| --- | --- | --- | --- | --- | --- | --- |
| 1 | Coordinator/volunteer welcomes group |  |  |  |  |  |
| 2 | Feedback from previous group / EVA experience |  |  |  |  |  |
| 3 | Session is introduced |  |  |  |  |  |
| 4 | Each activity introduced by volunteer/ coordinator, according to manual |  |  |  |  |  |
| 5 | Participants given opportunity to participate in group discussions |  |  |  |  |  |
| 6 | Groups split for activities/smaller group discussion |  |  |  |  |  |
| 7 | Challenge tasks introduced by coordinators/ volunteers |  |  |  |  |  |
| 8 | Equipment and resources described in manual have been added to board |  |  |  |  |  |
| 9 | Any problems between participants in group are addressed by coordinator/volunteer |  |  |  |  |  |
| 10 | Coordinator/volunteer uses total communication to support problems with communication |  |  |  |  |  |
| 11 | When real word problems are shared in the group, they are discussed in group |  |  |  |  |  |
| 12 | Coordinator/ volunteers check understanding of participants during session , e.g. before tasks |  |  |  |  |  |
| 13 | Example of modelling by coordinator/volunteers |  |  |  |  |  |
| 14 | Coordinator/ volunteers ask open questions |  |  |  |  |  |
| 15 | Coordinator/ volunteers provide positive feedback |  |  |  |  |  |
| 16 | Coordinator/volunteers redirects group back to aims of session when the conversation goes off topic |  |  |  |  |  |
| 17 | Group discussion is summarized by coordinator/volunteer |  |  |  |  |  |
| 18 | Participants and/or coordinators share real world examples in group |  |  |  |  |  |
| 19 | Example of expression of positive emotion within the group e.g. people are laughing, telling jokes |  |  |  |  |  |
| 20 | Example of participants talking to each other without being prompted by coordinator/volunteers |  |  |  |  |  |
| 21 | Attempts are made to elicit positive comments within the group |  |  |  |  |  |
| 22 | Balance of contributions by participants within group i.e. impressionistically, no participant spoke more than 50% of the time during session |  |  |  |  |  |
| 23 | Session kept to time |  |  |  |  |  |
| 24 | Volunteers/ coordinators kept to session plan for majority of session |  |  |  |  |  |

Note: Shaded cells could not be scored as N/A

S3 Supporting Information: Unit Costs used in the Economic Assessment

| **Item** | **Unit** | **Unit cost**  **(£,2017/18 prices)** | **Notes/assumptions** | **Sources/references** |
| --- | --- | --- | --- | --- |
| Project manager time | Working hour | 65.79 | Assumes total annual employment costs of £102230. Calculation assumes a project manager paid on point 44 of national payscale (£49149 p.a.), 30% salary on-costs @ £14745 p.a. and 60% institutional overheads upon total salary@£38336. Excludes London Allowance. Working time assumed as 42 working weeks p.a/37 hours p.w. Note that the estimated cost is broadly equivalent to the economic cost of a Band 8a NHS community-based SLT principal (£63 per working hour). | 2017/18 National payscale information obtained from: University & College Union higher education single pay spine. https://www.ucu.org.uk/he_singlepayspine (accessed 12/03/19)  2017/18 Hourly cost for a NHS community-based SLT principal obtained from: Curtis L, Burns A (2018). Unit costs of health & social care, 2018. University of Kent, Canterbury. https://www.pssru.ac.uk/pub/uc/uc2018/community-based-health-care-staff.pdf (accessed 12/03/19) |
| Co-ordinator time | Working hour | 29.99 | Assumes total annual employment cost of £46612.  Calculation assumes a co-ordinator salary of £27581 p.a., 30% salary on-costs @ £8274 p.a. and 30% institutional overheads upon total salary @£10757. Working time assumed as 42 working weeks p.a/37 hours p.w. | Co-ordinator base salary obtained from: figures used in research funding proposal. |
| Volunteer time | Hour of leisure time | 6.86 | DoT document reports the perceived cost of non-working time for 'other' purpose to be £6.04 at 2010 prices.  2010 value uprated to 2017/18 prices using GDP inflator of 13.6% | Value of leisure time obtained from: Department for Transport (2014). Transport Analysis Guidance (TAG).  Values of time and vehicle operating costs TAG Unit 3.5.6. (Table 2). https://webarchive.nationalarchives.gov.uk/20140304110038/http://www.dft.gov.uk/webtag/documents/expert/pdf/U3_5_6-Jan-2014.pdf (accessed 12.03.19)  GDP inflation index obtained from: Curtis L, Burns A (2018). Unit costs of health & social care, 2018. University of Kent, Canterbury. https://www.pssru.ac.uk/pub/uc/uc2018/sources-of-information.pdf (accessed 12/03/19) |
| Laptop | Item/user | 135.00 | Purchase price assumed as £810 for a 'gaming' laptop (e.g. HP ProBook 450 2017). Per user cost assumed as 1/6 of full cost based on 6 month loan and 3-year life span. (Cost not annuitised) | Price obtained from: project proposal for expenditure. |
| Headset | Item/user | 3.33 | Purchase price assumed as £20. Per user cost assumed as 1/6 of full cost based  on 6 month loan and 3-year life span.(Cost not annuitised) | Price obtained from: project proposal for expenditure. |
| Ethernet cable | Item/user | 1.33 | Purchase price assumed as £8.Per user cost assumed as 1/6 of full cost based on 6 month loan and 3-year life span.(Cost not annuitised) | Price obtained from: broad estimate from scoping several major internet sales sites. |
| Dongle | Item/user | 5.00 | Purchase price was £5 and data price was £25. Per user cost assumed as 1/6 of full cost based on 6 month loan and 3-year life span.(Cost not annuitised) | Price obtained from: project record of expenditure. |
| Software hosting | Group | 38.25 | Last bill paid by project = 435.60 Euros for one year. This is equivalent to £383 per year (or £31.92 per month) based on an average 2017/18 currency conversion rate of 0.8793. Assuming that a group runs for 6 months, and uses only 20% of the software's capacity, the total cost per group = £38. | 2017/18 Currency conversion rate obtained from: HM Revenue & Customs (2019). HMRC foreign exchange rates:  yearly averages and spot rates. Average for the year to 31 March 2018. https://www.gov.uk/government/uploads/system/uploads/attachment_data/file/696926/average-year-to-march-2018.csv/preview (accessed 23 March 2019) |
| IT expert time | Working hour | 53.20 | Actual hourly rate paid by project = 50 euros plus VAT (2017/18 standard VAT verified as being the same as 2019 rate of 21%). This is equivalent to £53.20 per hour based on an average 2017/18 currency conversion rate of 0.8793. | Time/expenditure estimates obtained from: actual amounts paid by the project to the relevant individual for input to various interconnected projects and their retrospective approximation of time allocation to this project.  2019 Netherlands VAT rate obtained from: Government of the Netherlands. VAT rates and exemptions. https://www.government.nl/topics/vat/vat-rates-and-exemptions (accessed 23/03/19)  2017/18 Currency conversion rate obtained from: HM Revenue & Customs (2019). HMRC foreign exchange rates:  yearly averages and spot rates. Average for the year to 31 March 2018. https://www.gov.uk/government/uploads/system/uploads/attachment_data/file/696926/average-year-to-march-2018.csv/preview (accessed 23 March 2019) |

S4 Supporting Information: Detailed Cost Breakdown

| **INTERVENTION COMPONENT** | | **RESOURCES** | | | | | **COSTS** | | | | |
| --- | --- | --- | --- | --- | --- | --- | --- | --- | --- | --- | --- |
|  |  | **Description** | **Estimated quantities** | | | | **Total cost (£, 2017-18 prices)** | | | | |
|  |  |  | **North** | **East** | **South** | **West** | **North** | **East** | **South** | **West** | **Mean** |
| **1** | **TRAINING FOR COORDINATORS & VOLUNTEERS*** | |  |  |  |  |  |  |  |  |  |
| a | Trainer time | Total contact and non-contact time(hours) including travel | 44 | 19 | 42 | 32 | 2915 | 1265 | 2739 | 2079 |  |
| b | Coordinator time | Total contact and non-contact time(hours) including travel | 11 | 8 | 17 | 13 | 315 | 250 | 504 | 384 |  |
| c | Volunteer time | Total contact and non-contact time(hours) including travel | 29 | 29 | 39 | 80 | 196 | 201 | 264 | 551 |  |
| d | Trainer travel costs | Expenses incurred for travel to training |  |  |  |  | 1132 | 0 | 210 | 163 |  |
| e | Coordinator travel costs | Expenses incurred for travel to training |  |  |  |  | 34 | 12 | 37 | 130 |  |
| f | Volunteer travel costs | Expenses incurred for travel to training |  |  |  |  | 13 | 0 | 7 | 94 |  |
| g | Reading packs & handbooks | Total number of reading packs & handbooks issued | 4 | 2 | 13 | 13 | 20 | 10 | 65 | 65 |  |
| h | Other equipment/materials | Total number of other equipment/materials used | 2 | 0 | 0 | 0 | 2 | 0 | 0 | 0 |  |
|  | TOTAL COST FOR TRAINING | |  |  |  |  | 4627 | 1738 | 3826 | 3465 | 3414 |
| **2** | **PROJECT MANAGER INPUTS TO GROUPS**** | |  |  |  |  |  |  |  |  |  |
| a | Project manager time - general support to group | Total contact and non-contact time(hours) excluding travel | 13 | 5 | 6 | 4 | 853 | 297 | 396 | 281 |  |
| b | Project manager time - supporting coordinators | Total contact and non-contact time(hours) excluding travel | 3 | 2 | 4 | 2 | 215 | 154 | 253 | 154 |  |
| c | Project manager time - supporting volunteers | Total contact and non-contact time(hours) excluding travel | 3 | 2 | 3 | 2 | 226 | 116 | 182 | 154 |  |
| d | Project manager time - supporting participants | Total contact and non-contact time(hours) excluding travel | 8 | 3 | 5 | 2 | 517 | 187 | 303 | 132 |  |
| e | Project manager travel time | Time spent travelling related to this group (hours) | 0 | 2 | 3 | 3 | 0 | 99 | 220 | 198 |  |
| f | Project manager travel costs | Expenses incurred for travel related to this group |  |  |  |  | 25 | 5 | 63 | 108 |  |
|  | TOTAL COST FOR PROJECT MANAGER INPUTS TO GROUPS | |  |  |  |  | 1835 | 858 | 1416 | 1027 | 1284 |
| **3** | **COORDINATOR INPUTS TO GROUPS** | |  |  |  |  |  |  |  |  |  |
| a | Coordinator time | Total contact and non-contact time(hours) including travel | 87 | 49 | 61 | 74 | 2610 | 1470 | 1829 | 2220 |  |
|  | TOTAL COST FOR COORDINATOR INPUTS TO GROUPS | |  |  |  |  | 2610 | 1470 | 1829 | 2220 | 2032 |
| **4** | **VOLUNTEER INPUTS TO GROUPS** | |  |  |  |  |  |  |  |  |  |
| a | Volunteer time - supporting group participants online*** | Total contact and non-contact time(hours) | 56 | 84 | 112 | 112 | 384 | 576 | 768 | 768 |  |
| b | Volunteer time - supporting participants offline | Total contact and non-contact time(hours) | 54 | 39 | 2 | 20 | 372 | 268 | 10 | 135 |  |
| c | Volunteer travel time | Time spent travelling to support participants (hours) | 9 | 21 | 0 | 6 | 62 | 141 | 0 | 43 |  |
| d | Volunteer travel costs | Expenses incurred for travel |  |  | 0 |  | 11 | 49 | 0 | 71 |  |
|  | TOTAL COST FOR VOLUNTEER INPUTS TO GROUPS | |  |  |  |  | 829 | 1034 | 779 | 1018 | 915 |
| **5** | **HARDWARE** | |  |  |  |  |  |  |  |  |  |
| a | Laptop | Proportion of equipment purchase cost | 9 | 7 | 5 | 5 | 1215 | 945 | 675 | 675 |  |
| b | Headset | Proportion of equipment purchase cost | 4 | 3 | 2 | 2 | 13 | 10 | 7 | 7 |  |
| c | Ethernet cable | Proportion of equipment purchase cost | 9 | 9 | 8 | 8 | 12 | 12 | 11 | 11 |  |
| d | Dongle | Proportion of equipment purchase cost | 1 | 0 | 1 | 0 | 5 | 0 | 5 | 0 |  |
|  | TOTAL COST FOR HARDWARE | |  |  |  |  | 1245 | 967 | 697 | 692 | 901 |
| **6** | **SOFTWARE** | |  |  |  |  |  |  |  |  |  |
| a | IT staff time - set-up & maintenance***** | Total time (hours) | 17 | 17 | 17 | 17 | 901 | 901 | 901 | 901 |  |
| b | IT staff time - online support to groups****** | Total time (hours) | 9 | 9 | 9 | 9 | 477 | 477 | 477 | 477 |  |
| c | Software hosting cost | Estimated proportion of annual cost |  |  |  |  | 38 | 38 | 38 | 38 |  |
|  | TOTAL COST FOR SOFTWARE | |  |  |  |  | 1416 | 1416 | 1416 | 1416 | 1416 |
|  | **TOTAL COST FOR GROUP (inc. hardware)** |  |  |  |  |  | **£12,562** | **£7,483** | **£9,963** | **£9,838** | **£9,961** |
|  | **TOTAL COST FOR GROUP (exc. hardware)** |  |  |  |  |  | **£11,316** | **£6,516** | **£9,265** | **£9,146** | **£9,061** |
|  | **Average cost per participant (exc. hardware)** | Accounting for actual number of participants | 5 | 9 | 7 | 8 | **£2,263** | **£724** | **£1,324** | **£1,143** | **£1,364** |
|  | **Average cost per online attendance (exc. hardware)** | Accounting for actual attendance rates | 58 | 96 | 92 | 101 | **£195** | **£68** | **£101** | **£91** | **£114** |
|  |  |  |  |  |  |  |  |  |  |  |  |
| **SCENARIO ANALYSIS: total and average costs when trainer time and travelling costs are reduced for high-cost groups** | | |  |  |  |  |  |  |  |  |  |
|  | **TOTAL COST FOR GROUP (exc. hardware)** | Total cost when trainer time cost (1a) for  North/South/West Groups is reduced to £1265 (the value for the East Group) and trainer travel costs (1d) for North Group is reduced to £124 (the average for the other 3 groups) |  |  |  |  | **£8,658** | **£6,516** | **£7,791** | **£8,332** | **£7,824** |
|  | **Average cost per participant (exc. hardware)** | Accounting for actual number of participants | 5 | 9 | 7 | 8 | **£1,732** | **£724** | **£1,113** | **£1,041** | **£1,153** |
|  | **Average cost per online attendance (exc. hardware)** | Accounting for actual attendance rates | 58 | 96 | 92 | 101 | **£149** | **£68** | **£85** | **£82** | **£96** |

| * | Separate sessions for each group but with coordinators and volunteers within each group attending together. |
| --- | --- |
| ** | Participant suitability (in tech use and language) was mostly screened by project managers (54 screens@15 minutes each), except in North Group (13 screens) where it was done locally. This task would need to be absorbed by others in future roll-out scenarios. |
| *** | Includes 30 min online pre-meeting between co-ordinators & volunteers before each session |
| **** | We exclude associated costs for delivering/collecting equipment. |
| ***** | Estimated as 16 hours total to set up a new island on the server (so 4 hours per group) including accounts, creating portal, server settings; and 1 hour per week for weekly backup of the server and other technical maintenance (so 0.5 hours per group since 2 groups ran concurrently). |
| ****** | Estimated as 2-4 hours a month for online support to users (so 1.5 hours per group since 2 groups ran concurrently). |

S5 Supporting Information: Breakdown of Time Inputs to the Groups (excluding travel time; rounded to full hours)

|  | Number of hours |
| --- | --- |
| **Volunteers** |  |
| Online support to the overall group: mean per group, across 4 groups | 91 |
| Offline support to the overall group: mean per group, across 4 groups | 29 |
| Offline support to individuals: mean per participant, across 29 participants | 4 |
| **Coordinators** |  |
| Online and offline* support to overall group (contact and non-contact): mean per group, across 4 groups | 68 |
| **Researchers** |  |
| Offline support to overall group (contact and non-contact): mean per group, across 4 groups | 7 |
| Offline support to co-ordinators: mean per group, across 4 groups | 3 |
| Offline support to volunteers: mean per group, across 4 groups | 3 |
| Offline support to participants: mean per group, across 4 groups | 4 |

*Questionnaires for coordinators about their time inputs did not ask them to differentiate between online and offline time
