# Supplementary material for: A randomised trial of social support group intervention for people with aphasia: A Novel application of virtual reality
Source: PLoS One. 2020 Sep 24;15(9):e0239715. doi: 10.1371/journal.pone.0239715 (PMC7514104; doi:10.1371/journal.pone.0239715)
Supplement: S1 Protocol — (DOCX) [file pone.0239715.s003.docx]

Delivering group support for people with aphasia through a virtual communication environment

Study Protocol

**Background**

Between a quarter [1] and one third [2] of stroke survivors acquire a lasting aphasia, with profound consequences for the person’s quality of life [3]. Aphasia reduces individuals’ social networks [4, 5], community integration [6], satisfaction with life [7], social activities [8] and maintenance of friendships [9]. Support groups may counter some of these effects, e.g. by helping to build relationships and re-connecting individuals with their families, friends and local community [10, 11]. Evidence for the benefits includes self-reports from service users [12, 13] and results of evaluation studies [4, 14, 15]. A recent systematic review concluded that community and outpatient group interventions for people with aphasia can improve specific linguistic processes (when these are targeted) and benefit social networks and community access [16].

Support groups often feature in aphasia provision both in the UK and internationally [17]. However, access to such services is far from universal. There is evidence that users of support groups tend to live close to the venue or be able to travel independently [18], suggesting that the groups may not reach people in remote areas, or with mobility problems. Indeed many stroke survivors experience low levels of community provision [19]. Wider access to support may be promoted by alternative models of service delivery, and particularly by the use of remote digital technologies. Such technologies have been explored in the context of aphasia assessment [20, 21] and therapy [22, 23] and have been used to support people with other conditions [e.g. 24].

A technology that has the potential to deliver remote group support is EVA Park. This is a virtual island created with and for people with aphasia [25]. It can be populated by several people at the same time. Users communicate with each other via speech, using a head set and microphone. Our first study [26] showed that Eva Park can deliver one to one language stimulation, with gains on a standardised measure of functional communication. Feasibility findings were good, with no attrition, strong compliance with intervention and high acceptability to users. This project will explore the potential of EVA Park to deliver remote group support. We will explore this in the context of a ‘typical’ model of care, which in the UK involves voluntary provision.

**Study Aims and Questions**

This project aims to explore the feasibility and acceptability of a remote support group intervention for people with aphasia delivered through EVA Park. Specifically we will: Explore and document the processes involved in delivering the intervention; Explore the suitability of EVA Park for delivering this type of intervention; Investigate the acceptability of the intervention, and barriers and facilitators, from the perspective of those who receive the intervention, those who deliver the intervention and, where relevant, family members or care givers; Investigate the impact of the intervention on wellbeing, communication, social connectedness, language and quality of life; Examine the costs of the intervention.

The study questions are:

Is delivery of group social support to people with aphasia feasible via EVA Park, as indicated by the recruitment and retention of participants, compliance with intervention, and fidelity assessments of intervention?

Does a programme of group social support delivered in EVA Park improve scores on measures of well-being, communication, social connectedness, language and quality of life?

Is delivery of group social support via EVA Park acceptable to those who deliver and receive the intervention, and to care givers? What, if any, barriers do they experience and what are the perceived benefits?

How do people with aphasia experience group social support in EVA Park, as assessed by Human Computer Interaction (HCI) observations and interviews?

What is the cost of providing a non-intensive programme of group social support delivered in EVA Park?

**Methods**

***Design***

The study will employ a randomised, waitlist controlled design. Four groups will be recruited, each comprising a co-ordinator, four volunteers and eight or nine people with aphasia. Two groups will be randomised to an immediate condition, and will receive intervention in months 6 – 11 of the study. The other two groups will be randomised to a delayed condition and will receive intervention in months 13 – 18. Outcome measures will be administered to participants with aphasia in months 5 (T1), 12 (T2) and 19 (T3). Thus, participants in the immediate condition will be tested before intervention, after intervention and at 6 months follow up. Those in the delayed condition will be tested twice before intervention and once after intervention.

***Ethical approval***

Ethical approval to conduct the study has been granted by Ethics Committee of the School of Health Sciences, City, University of London (Language and Communication Science Proportionate Review) LCS/PR/Staff/16-17/06. The trial sponsor is City, University of London, and the study is funded by the Stroke Association (TSA 2016/5).

***Participants***

Four participant groups will be recruited comprising: a co-ordinator, four volunteers, and eight or nine people with aphasia. Groups will be in different geographical locations, both within and outside London.

Co-ordinators will have experience in leading social support groups for people with aphasia within the voluntary sector. They will be renumerated for their involvement. They will be identified through the Stroke Association and other relevant networks. Volunteers will have experience in supporting social groups for people with aphasia and/or other aspects of aphasia intervention. They will be linked to co-ordinators’ existing groups or recruited specifically for the study, e.g. via City University student cohorts. Co-ordinators and volunteers will deliver the intervention in the study.

Participants with aphasia will be stroke survivors with moderate/mild aphasia and no co-existing diagnosis affecting cognition, such as dementia. They will not have severe hearing or visual impairments and will have been fluent in English before the stroke. These criteria will rule out individuals who are unlikely to benefit from the intervention. For example, communication in EVA Park is largely speech dependent, making it unsuitable for those with very severe aphasia. Similarly, severe sensory impairments make use of EVA Park very difficult.

***Recruitment and Consent Processes***

Participants with aphasia will be identified via diverse routes. Most will be referred by the co-ordinators, e.g. from their current social groups. We will also accept referrals from community groups that are not involved in the study. We will particularly target individuals who are known to the co-ordinators but unable to access face to face services. Some participants may self-refer, having learnt about the project from the website, social media or word of mouth. We may also contact individuals with aphasia known to the University who have expressed an interest in being involved in our research.

Participants will be screened by the project managers. Eligibility will be established via the Frenchay Aphasia Screening Test [27] and a screening questionnaire, covering the history of the stroke, language background, hearing, vision, and any co-morbidities.

All participants will give informed written consent. Materials for the participants with aphasia have been designed following accessibility guidelines for people with aphasia [28]. Consent will be obtained by the project managers, who are qualified speech and language therapists. They will read and explain the information and consent materials, and respond to participants’ questions. Participants’ capacity to give consent will be judged by the project managers and, if relevant, by the referring co-ordinators. If necessary yes/no questions will be used to verify participants’ understanding of the consent materials. Participants will not be consented if capacity is in doubt.

***Randomisation***

Randomisation of groups to the immediate or delayed condition will be conducted via a computer randomisation tool, and will take place before participants with aphasia are recruited. Participants with aphasia will be assigned to groups according to their geographical location.

***Intervention***

The intervention is described in Table 1, Following the TIDieR Checklist [29]

| 1. Name | Full Title: Group Social Support Delivered in EVA Park  Short title: EVA Park Aphasia Groups |
| --- | --- |
| 2. Rationale, Theory or Goal of the Intervention | Intervention aims to counter the negative impacts of aphasia on quality of life, and to facilitate living well with aphasia [30]. Activities aim to promote wellbeing, give participants experiences of communicative success and foster social connection. Intervention will draw on the principles of asset based intervention [31] and positive psychology [32], for example by encouraging reflection of personal strengths and problem solving capabilities. Exploring issues of personal identity will also be focussed, given the impact of aphasia on a person’s sense of self [33] |
| 3. Materials | Intervention is described in a manual, which outlines the underlying principles, group treatment techniques and provides detailed session plans. Resources for individual sessions will be provided in EVA Park; for example these may comprise screen displays of relevant images or videos for the group to watch. Challenge tasks will be set between sessions. For example, group members might be invited to meet up in EVA Park and share experiences in which they took a risk. |
| 4. Therapy Procedures | Therapy involves group sessions in EVA Park addressing personal or topical themes. Personal themes are: you, aphasia, resilience and personal strengths. Topical themes are: music, art, literature, comedy, eating out and sports day. Themes are addressed through discussion, tasks and assignments. For example, under the theme of ‘you’, participants share 5 personal characteristics that mark them as individuals, discuss their avatar in EVA Park and consider how it is like/unlike their real selves. Under comedy the group watch clips of famous TV comedies, discuss what makes them funny and share amusing personal stories. Challenge tasks are set between sessions that further address the theme. For example the ‘you’ task involves meeting another group member in EVA Park outside the scheduled session to share personal attributes. The remaining sessions address a group project which involves generating a meaningful output about aphasia (such as a film, or information leaflet). This engages problem solving skills and requires group members to draw on their individual strengths in realising a collective goal. All themes encourage positive reflection: what are my strengths? What gives me pleasure? What do I find funny? |
| Intervention Providers | Intervention is led by the co-ordinators and supported by the group volunteers. |
| Mode of Delivery | All sessions involve a mix of whole group activities and tasks in which the group divides into smaller sub-groups. Between session challenge tasks are self-directed and usually in pairs |
| Location | All sessions take place in EVA Park, a virtual island designed with and for people with aphasia (Wilson et al, 2015). EVA Park contains a number of functional and fantastic locations, in which users can meet and converse. These include: houses, a café, a health centre, a tree house, a bar and tardis. Users are represented by personalised avatars and communicate in real time mainly via speech (there is also a message typing facility). There are many areas suitable for group meetings, such as a lakeside set of reclining chairs. The environment is amusing and designed to stimulate conversation. For example there are attractive green spaces, wild life and fun features such as a disco with a dance ball. Participants will access EVA Park using a computer in their own home (if necessary they will be loaned hardware). EVA Park will be set up on their computers by the co-ordinators or volunteers, who will also train them in how to use the platform. Co-ordinators and volunteers will access EVA Park from a home computer or from a computer in their community centre. |
| When and how much | Intervention will take place over a period of 6 months. In this time groups will meet every two weeks for 90 minutes each session (14 sessions in all). |
| Tailoring | Although session topics are fixed, all session plans allow participants to introduce personally relevant and selected materials. For example, in the music session participants share and discuss personally selected clips of music. Discussions are responsive to the issues raised by group members and their interests. The group project will be chosen separately by each group. Tailoring will also take account of the varying communication skills and strategies employed by group members. For example, some may be able to contribute extended spoken discourse, while others will need to respond to structured or even closed questions. |
| Adherence and Fidelity | Adherence to the planned therapy will be promoted through training and supervision of the co-ordinators and volunteers (see separate section). Adherence to the regime will be monitored by recoding the number of sessions delivered and attended in each group. Automatic computer logging will record the amount of time participants spend in EVA Park both during and outside scheduled sessions.  Fidelity of treatment delivery will be assessed by recoding a selection of sessions and scoring them against a checklist of core intervention components (see details under Fidelity Measures). |

***Training and Supervision of the Intervention Providers***

Group co-ordinators and volunteers will be provided with two, 3-hour face-to-face training sessions before intervention begins. Each team will be trained separately by the project managers. The first session will cover technical aspects including set-up of EVA Park, access, avatar creation and customisation, navigation, troubleshooting and providing remote technical support to people with aphasia. The second session will provide training on delivering the intervention. This will include direction and practice in managing a group in a virtual environment. Specific advice will be given on how to employ supportive communication techniques in EVA Park, e.g. how to use pointing, giving time, reflective listening, using message writing to support understanding, and using visual cues. Training will be provided at the University or in the co-ordinator’s community setting, according to the needs of participants.

In addition to training, the research managers will provide monthly supervision sessions (1 hour) to co-ordinators and volunteers throughout the 6 month intervention period. These supervision sessions will take place online, in a private area of EVA Park that is not accessible to all users. They will review progress, and respond to the concerns raised by each group. It is envisaged that most time will be spent brainstorming problems and discussing how to support individual group members. Ad hoc support will also be available outside these sessions, e.g. for technical difficulties. This will be provided by phone, email and virtual contact in EVA Park.

***Feasibility Measures***

Feasibility of recruitment and retention will be assessed by reporting the number of participants who are eligible from those screened and the consent rate of those eligible to take part. Rates and reasons for attrition will be recorded.

Compliance with intervention will be assessed by recording the number of sessions delivered by each group, and individual attendance at those groups. Computer logs will record the amount of time spent by each participant in EVA Park.

To assess fidelity, a checklist of core intervention components will be developed. These will be derived from the manual and from discussion with the project managers. Thirty treatment sessions will be recorded, using screen capture technology. Recordings will span the 4 treatment groups and will be taken from early and late in the intervention. At least 15 of these (25% of intervention sessions) will be assessed using the checklist. Raters will be RAs who are not otherwise involved in the study. Five recordings will be assessed by both raters to evaluate interrater reliability.

***Outcome Measures***

Primary outcome measures:

Warwick-Edinburgh Mental Well-being Scale (WEMWBS) [34]. This scale was developed to monitor mental wellbeing in the general population and to evaluate intervention effects on mental wellbeing. It comprises 14, positively worded statements, such as ‘I’ve been feeling good about myself’, that have to be rated on a 5 point scale. These are summed, yielding a single wellbeing score (/70).

Communication Activities of Daily Living-2 (CADL-2) [35]. This is a standardised assessment of everyday communication for people with aphasia, which is based on specific scenarios, such as going to the doctor. The CADL-2 contains 50 items scored 2, 1 or 0. Scoring credits communicative success rather than the use of formally correct language. The total score will be analysed.

Secondary measures:

Social Connectedness Scale-Revised (SCS-R) [36]. This consists of 20 positively or negatively worded statements, such as ‘I feel close to people’ and ‘I see myself as a loner’, that have to be rated on a six point scale. All items are summed (with reverse scores for negative items) to produce a single score (/120).

Western Aphasia Battery-Revised (WAB-R) [37]. This is a standardised language assessment designed for people with aphasia. Only sections assessing speech production and comprehension will be administered. These produce a single aphasia quotient score that will be analysed.

Stroke and Aphasia Quality of Life-39 (SAQOL-39) [38] In this assessment 39 items, such as asking how much trouble someone had speaking in the last week, must be rated on a five- point scale. Three domains are covered: physical, psychosocial and communication, yielding mean domain and total scores. The mean total will be analysed (/5).

All measures have strong psychometric qualities and have shown sensitivity to therapy induced change. Three (CADL-2 [35], WAB-R [37] and SAQOL-39 [38]) have been widely used in previous trials of aphasia therapy [39]. Two measures (WEMWBS [34] and SCS-R [36]) were not designed for people with aphasia. However, they are brief and make limited language demands.

Outcome measures will be administered face to face (not in EVA Park) by blinded assessors (see below). It is envisaged that most testing will take place in participants’ homes, although some may be assessed in their local community centre.

***Blinding***

Outcome measures will be administered by research assistants (RAs) who will be blinded to time point, and whether participants are in the immediate or delayed condition. To protect blinding we will ask participants not to tell the RAs whether they have received EVA Park intervention. Visits will be scheduled by the project managers and testing RAs will not have access to participant files or to any details that could potentially unblind them. We will ask testing RAs to indicate if they have become unblinded, and, if relevant, subsequent testing will be carried out by a different RA.

***Acceptability Measures***

Semi-structured interviews will be conducted with all participants with aphasia post intervention to determine their views about the intervention, any perceived benefits and/or negative factors. Half the participants from the immediate condition will be interviewed 6 months after the end of intervention to explore any perceived long term changes. We will aim to interview 10 family members/caregivers of participants (convenience sampling) to explore their perceptions of the intervention, and any perceived benefits/negative factors. Implications for carer burden will also be explored.

All co-ordinators and at least 8 volunteers will be interviewed to explore their perceptions of delivering social support groups in EVA Park. Four consensus discussions will be held with the co-ordinators and volunteers from each group in order to identify benefits, challenges and potential improvements that could be made to the intervention.

Interviews and consensus discussions will be conducted by a qualitative researcher who is not otherwise involved in the study. Participants with aphasia will be interviewed in their home or community centre. Family members may also be interviewed at home or remotely (e.g. on Skype). Co-ordinators and volunteers will be interviewed in their community centre or remotely. Consensus discussions will take place in the local community centre or in the University.

***Exploring the Experience of EVA Park Social Support Groups***

A convenience sample of 16 participants will take part in Human Computer Interaction (HCI) assessments. We will ensure that the sample spans all groups and that participants from the immediate and delayed condition are represented. Participants will be observed, at home, while they take part in a group session in EVA Park. The session will also be filmed using screen capture. Post session interviews will be conducted in order to explore participants’ feelings about the session and the degree to which they felt socially connected to others in their group. Participants involved in HCI data collection will each be visited twice, once early and once late in the intervention. This will enable us to explore any changes in their experience over time.

HCI data collection and analysis will be conducted by a researcher who is not involved in any other aspect of the study.

***Economic Measures***

Data will be collected on all resources associated with intervention delivery, including training, supervision, support given by the project managers, hosting and technical support for EVA Park, hardware (such as computer loans to participants), time and travel expenses associated with setting up EVA Park in each participants’ home, and the time and travel expenses associated with inputs from co-ordinators and volunteers. Data will be collected via online or paper-based forms to be completed by project managers, co-ordinators and volunteers from all groups.

***Data Management and Analysis***

Paper files will be stored at City, University of London. Identifiable information will be separated from other data and kept in a locked filing cabinet. Anonymised, electronic data will be stored on a shared, drive, which is accessible only to designated staff members. Identifiable electronic data will be password protected.

Feasibility measures will be subject to descriptive statistical analysis. Descriptive data will be presented for all outcome measures at T1, T2 and T3 (means, standard deviations). An Intention to Treat analysis will be conducted on data from T1 and T2 to determine any indicative effects of treatment (time x condition ANOVA). A per protocol analysis on pooled data from all participants will explore pre to post intervention changes (repeated measures t tests or non parametric equivalents).

Interview and consensus discussion data will be recorded and transcribed verbatim. Framework analysis will be used to identify themes in the data and synthesise findings.

Sessions recorded for HCI analysis will be coded in order to quantify key features of participants’ experience. For example, instances of positive affect and social connection will be recorded (see [40] for an example of the methodology).

Relevant unit cost estimates will be attached to resource use data to determine the cost of delivering the intervention by each group, the average cost per group, average cost per participant and average cost per scheduled attendance. Sensitivity analyses will explore estimates under alternative assumptions, and factors influencing the cost of delivery, such as the number of hardware loans, will be identified

***Dissemination***

A dissemination event will be staged at City, University of London, in order to communicate the findings to participants, clinicians and the wider aphasia community. We will create a film about the project which will be made publicly available on YouTube.

Results will be reported at academic and clinical conferences, such as the International Aphasia Rehabilitation Conference, the conference of the British Aphasiology Society and the conference of the Royal College of Speech and Language Therapists.

At least three peer reviewed publications will be submitted reporting Outcomes (feasibility data, outcome measure data, cost data); Acceptability findings (interview and consensus discussion data) and HCI findings.

**References**

1. Ali, M. et al. (2015). Aphasia and dysarthria in acute stroke: recovery and functional outcome. *International Journal of Stroke*, *10*, 400-406.

2. Dickey, L. et al. (2010). Incidence and profile of inpatient stroke-induced aphasia in Ontario, Canada. *Archives of Physical Medical Rehabilitation*, *91*, 196-202.

3. [Hilari, K.](http://openaccess.city.ac.uk/view/creators_id/k=2Ehilari.html) (2011). The impact of stroke: Are people with aphasia different to those without? Disability and Rehabilitation, *33*(3), 211-218.

4. Vickers, C. (2010) Social networks after the onset of aphasia: The impact of aphasia group attendance. *Aphasiology*, *24*, 902–913.

5. Northcott et al. (2016). What factors predict who will have a strong social network following a stroke? *Journal of Speech Language and Hearing Research,* 59, 772-783*.*

6. Lee et al, (2015). Community integration and quality of life in aphasia after stroke. *Yonsei Medical Journal*, *56*(6), 1694-1702.

7. Nagayoshi, M. et al. (2008). Factors associated with life satisfaction in Japanese stroke outpatients. Disability and Rehabilitation, *30*(3), 222-30.

8. Cruice, M. et al. (2006). Quantifying aphasic people’s social lives in the context of non-aphasic peers. *Aphasiology, 20*, 1210-1225.

9. Northcott, S., & Hilari, K. (2011). Why do people lose their friends after a stroke? *International Journal of Language and Communcation Disorders, 6*(5), 524-534.

10. Elman, R. (2010). The increasing popularity of aphasia groups: some reasons why. *Perspectives On Neurophysiology & Neurogenic Speech & Language Disorders, 20*(4), 120-124.

11. Carozza, L. & Shafi, N. (2013). Quality of life in aphasia community group members: A social model of clinical treatment. *Acta Neuropsychologia, 11*(1), 1–7.

12. Legg et al. (2007). Volunteer Stroke Service (VSS) groups for patients with communication difficulties after stroke: a qualitative analysis of the value of groups to their users. *Clinical Rehabilitation, 21*, 794-804.

13. Kong, A. (2011). Family members’ report on speech-language pathology and community services for persons with aphasia in Hong Kong. Disability and Rehabilitation, *33*, 2633-2645.

14. Van de Gaag (2005). Therapy and support services for people with long-term stroke and aphasia and their relatives: A six-month follow-up study. *Clinical Rehabilitatiol*,*19*, 372-80.

15. Ross, A. et al. (2006). Evaluation of communication, life participation and psychological well-being in chronic aphasia: The influence of group intervention. *Aphasiology, 20*, 427-448.

16. Lanyon, L. et al. (2013). The efficacy of outpatient and community-based aphasia group interventions: A systematic review. *International Journal of Speech Language Pathology, 15*, 359-374.

17. Rose, M. et al. (2014). Aphasia rehabilitation in Australia: Current practices, challenges and future directions. *International Journal of Speech Language Pathology*, *16*(2), 169-180.

18. Code et al. (2001). Profiling the membership of self-help groups for aphasic people. *International Journal of Language and Communication Disorders*, *36*, 41-5.

19. Care Quality Commission (2011). Supporting Life after Services National Report. A review of services for people who have had a stroke and their carers. Available from: <http://www.cqc.org.uk/file/4349> .

20. Theodoros D, et al. (2008). Assessing acquired language disorders in adults via the Internet. *Telemed Journal of E Health, 14*, 552-559. 12.

21. Hill A, et al. (2009). The effects of aphasia severity on the ability to assess language disorders via telerehabilitation. *Aphasiology, 23*, 627-642.

22. Cherney LR, & Van Vuuren S. (2012). Tele rehabilitation, virtual therapists, and acquired neurologic speech and language disorders. *Seminars in Speech and Language, 33*(3), 243-257.

23. Woolf, C. et al. (2015). A comparison of remote therapy, face to face therapy and an attention control intervention for people with aphasia: A quasi-randomised controlled feasibility study. *Clinical Rehabilitation*, DOI: 10.1177/0269215515582074.

24. Best, B. & Butler, S. (2014). Virtual space: Creating a place for social support in second life. *Space Cult*, DOI1177/1206331213512235.

25. Wilson, S, et al. (2015). Codesign for People with Aphasia through Tangible Design Languages. *CoDesign, 11*(1), 21-34. doi: 10.1080/15710882.2014.997744

26. Marshall et al. (2016). Evaluating the benefits of aphasia intervention delivered in virtual reality: Results of a quasi-randomised study. *PLOS One*, 11(8): e0160381. doi:10.1371/journal.pone.0160381

27. Enderby P, et al. (1997). *Frenchay Aphasia Screening Test*. Oxford: Whurr Publishers.

28, Pearl, G. & Cruice., M. (2017) Facilitating the involvement of people with aphasia in stroke research by developing communicatively accessible research resources. *Topics in Language Disorders*, 2017; 37: 67-84.

29. Hoffmann T. et al (2014). Better reporting of interventions: template for intervention description and replication (TIDieR) checklist and guide *BMJ*, 348 <https://doi.org/10.1136/bmj.g1687g1687>

30. Brown K. et al (2012) Living successfully with aphasia: a qualitative meta-analysis of the perspectives of individuals with aphasia, family members, and speech-language pathologists. *International Journal of Speech and Language Pathology*, 14, 141–155.

31. Rippon S. et al. (2015) Head, hands and heart: asset-based approaches in health care. A review of the conceptual evidence and case studies of asset-based approaches in health, care and wellbeing. *The Health Foundation*, www.health.org.uk

32. Seligman M. et al. (2005) Positive psychology in progress. Empirical validation of interventions. *American Psychologist*, 60, 410–421.

33. Shadden B. (2007) Rebuilding identify through stroke support groups: Embracing the person with aphasia and significant others. In R. Elman (ed) *Group treatment of neurogenic communication disorders: The expert clinician’s approach* (2nd edition), 111-126. San Diego CA: Plural

34. Tennant R. et al. (2007) The Warwick-Edinburgh mental well-being scale (WEMWBS): development and UK validation. *Health and Quality of Life Outcomes*, 5(1), 63

35. Holland A. et al. (1999) Communication Activities of Daily Living-2. 1999; Austen TX: Pro-Ed

36. Lee R. et al. (1995) Measuring belongingness: The Social Connectedness and the Social Assurance Scales. *Journal of Counselling Psychology*, 42, 232-241

37. Kertesz A. (2006) Western Aphasia Battery-Revised. Pearson.

38. Hilari, K. et al. (2009). Psychometric properties of the Stroke and Aphasia Quality of Life Scale (SAQOL-39) in a generic stroke population. *Clinical Rehabilitation*, *23*(6), 544-557.

39. Brady M. et al. (2016) Speech and language therapy for aphasia following stroke. *Cochrane Database of Systematic Reviews*, Issue 6: Art. No.: CD000425. doi: 10. 1002/14651858.CD000425.pub4

40. Galliers J. et al. (2017) Experiencing EVA Park, a Multi-User Virtual World for People with Aphasia. *ACM Transactions on Accessible Computing*,10, 4, Article 15. https://doi.org/10.1145/3134227
